# Multigene Phylogeny and Morphology Reveal Unexpectedly High Number of New Species of *Cantharellus* Subgenus *Parvocantharellus* (Hydnaceae, Cantharellales) in China

**DOI:** 10.3390/jof7110919

**Published:** 2021-10-28

**Authors:** Ming Zhang, Chao-Qun Wang, Bart Buyck, Wang-Qiu Deng, Tai-Hui Li

**Affiliations:** 1 State Key Laboratory of Applied Microbiology Southern China, Guangdong Provincial Key Laboratory of Microbial Culture Collection and Application, Institute of Microbiology, Guangdong Academy of Sciences, Guangzhou 510070, China; zhangming_1985@163.com (M.Z.); dayangtutu@163.com (C.-Q.W.); dengwq@gdim.cn (W.-Q.D.); 2 Institut Systématique, Evolution, Biodiversité (ISYEB), UMR 7205, Muséum National d’Histoire Naturelle, Sorbonne Université, CNRS, Case Postale 39, 12 rue Buffon, F-75005 Paris, France; bart.buyck@mnhn.fr

**Keywords:** chanterelles, molecular phylogeny, morphology, seven new taxa, taxonomy

## Abstract

The genus *Cantharellus*, commonly known as chanterelles, has recently been divided into six subgenera; however, wider sampling approaches are needed to clarify the relationships within and between these groups. A phylogenetic overview of *Cantharellus* subgenus *Parvocantharellus* in China was inferred based on the large subunit nuclear ribosomal RNA gene (nrLSU), the DNA-directed RNA polymerase II subunit 2 (*rpb*2), and the transcription elongation factor 1-alpha (*tef*1). A total of nine species from China were assigned to the subgenus, including seven novel species, namely *Cantharellus* *aurantinus*, *C. austrosinensis*, *C. galbanus*, *C. luteolus*, *C. luteovirens*, *C. minioalbus*, and *C. sinominior*, and two known species, namely *C. albus* and *C. zangii*. The detailed descriptions and illustrations were provided based on the newly obtained data, with the comparisons to closely related species. *C. zangii* was restudied based on the paratype specimens and multiple new collections from the type locality. Futhermore, the Indian species *C. sikkimensis* was identified as a synonym of *C. zangii* based on the morphological and molecular analyses. A key to the Chinese species belonging to the subg. *Parvocantharellus* is also provided.

## 1. Introduction

*Cantharellus* Adans. ex Fr., typified by *C. cibarius* Fr., is an important genus of wild edible mushrooms and is renowned for its high culinary, economic, and ecological value. Chantarelles have a global distribution and are especially rich in subtropical to tropical zones, demonstrating ectomycorrhizal associations with a variety of plants [1]. Approximately 300 species have been estimated to exist worldwide, and nearly 180 species have been described thus far [1,2]. *Cantharellus* species possess a colourful pileus, nearly smooth to evidently lamellate-folded hymenophore with variously forked or anastomosing veins, and smooth basidiospores [1,3,4]. It is easy to recognise *Cantharellus* species at the genus level in the field solely based on their morphological features. However, the determination of their taxonomic positions at the species level is extremely complicated owing to overlaps in phenotypic variation among species. With the development of molecular biology, molecular-based studies have provided a basis for species identification and taxonomic development, especially for the molecular review of some type specimens and for re-classification of some old species based on new collections, so as to make species recognition more effective and accurate [5,6,7,8,9,10]. Molecular phylogenetic studies have delimited several species and revealed an unexpected species diversity. The *tef*1 gene has been identified as a suitable DNA barcoding marker to determine terminal relationships among closely related *Cantharellus* species [1,6,11,12,13,14].

Recently, within the genus *Cantharellus*, six subgenera (subg. *Afrocantharellus* Eyssart. & Buyck, subg. *Cantharellus* Adans. ex Fr., subg. *Cinnabarinus* Buyck & V. Hofst., subg. *Parvocantharellus* Eyssart. & Buyck, subg. *Pseudocantharellus* Eyssart. & Buyck, and subg. *Rubrinus* Eyssart. & Buyck) were proposed based on a phylogenetic analysis of widely distributed samples [1]. The subgenus *Parvocantharellus*, typified by *C. romagnesianus* Eyssart. & Buyck, was described as a monophyletic assemblage of mostly markedly small, yellow, orange, pink, or red species, presenting with a lilac-purple or brownish tinge in certain cases, particularly in the pileus centre, and exhibiting principally thin-walled hyphal endings and abundant clamp connections [1,15]. Species in subg. *Parvocantharellus* are mainly distributed in the northern hemisphere [16].

The names of the European and American species, such as *C. cibarius* Fr., *C. cinnabarinus* Schwein.) Schwein., and *C. minor* Peck, are often misapplied in Chinese samples, and a large number of undescribed taxa exist in China [17,18]. Thus far, only the following 10 species have been originally described from China: *C. albus* S.P. Jian & B. Feng, *C. hainanensis* N.K. Zeng, Zhi Q. Liang & S. Jiang, *C. hygrophoroides* S.C. Shao, Buyck & F.Q. Yu, *C. macrocarpus* N.K. Zeng, Y.Z. Zhang & Zhi Q. Liang, *C. phloginus* S.C. Shao & P.G. Liu, *C. tuberculosporus* M. Zang, *C. vaginatus* S.C. Shao, X.F. Tian & P.G. Liu, *C. versicolor* S.C. Shao & P.G. Liu, *C. yunanensis* W.F. Chiu, and *C. zangii* X.F. Tian, P.G. Liu & Buyck [2,19,20,21,22,23,24,25,26,27]. 

In our survey of the species diversity of *Cantharellus* in China, we discovered some distinct *Cantharellus* samples. The subsequent morphological and molecular analyses of their *tef*1 and LSU + *tef*1 + *rpb*2 gene sequences confirmed that these samples belong to the subg. *Parvocantharellus*, representing nine independent species, including seven new species, which are described and illustrated herein.

## 2. Materials and Methods

### 2.1. Morphological Studies

Photographs of the basidiomata were taken in the field before they were collected. The macro-morphological descriptions were based on field notes and colour photographs. The colour codes that were used followed Kornerup and Wanscher [28]. The microscopic measurements were carried out on dried tissue sections that were stained with 5% KOH and 1% aqueous Congo red under a light microscope (Olympus BX51, Tokyo, Japan) with magnification up to 1000×. For basidiospore descriptions, the abbreviation [n/m/p] denotes n spores measured from m basidiomata of p collections; the notation (a–)b–c(–d) describes the basidiospore dimensions, where the range ‘b–c’ represented 90% or more of the measured values and ‘a’ and ‘d’ are the extreme values; L_m_ and W_m_ indicate the average length and width (±standard deviation) of the measured basidiospores, respectively; the Q value refers to the length/width ratio of an individual basidiospore; and Q_m_ refers to the average Q value of all of the measured basidiospores ± standard deviation. All of the line-drawings of the microstructures were made free-hand and were based on the rehydrated materials. The studied specimens were deposited in the Fungarium of Guangdong Institute of Microbiology (GDGM).

### 2.2. DNA Extraction, PCR Amplification and Sequencing

The genomic DNA was extracted from the voucher specimens using the Sangon Fungus Genomic DNA Extraction kit (Sangon Biotech Co., Ltd., Shanghai, China) according to the manufacturer’s instructions. Primer pairs LR0R/LR7 [29], tef1F/tef1R or tef-1Fcanth/tef-1Rcanth [1,30] and RPB2-5FCanth/RPB2-7cRCanth [1] were used to amplify the LSU, *tef*1, and *rpb*2 genes, respectively. The PCR reactions were performed in a total volume of 25 μL containing 0.5 μL template DNA, 11 μL distilled water, 0.5 μL of each primer, and 12.5 μL PCR mix [DreamTaqtm Green PCR Master Mix (2×), Fermentas, USA]. The amplification reactions were performed in a Tprofessional Standard Thermocycler (Biometra, Göttingen, Germany) under the following conditions: 95 °C for 4 min; then 35 cycles of denaturation at 94 °C for 60 s, annealing at 53 °C (LSU) /50 °C (*tef*1) /52 °C (*rpb*2) for 60 s, and extension at 72 °C for 60 s; with a final extension at 72 °C for 8 min. The PCR products were electrophoresed on a 1% agarose gel with known standard DNA markers and the sequencing was performed on an ABI Prism^®^ 3730 Genetic Analyzer (PE Applied Biosystems, Foster, CA, USA) at the Beijing Genomic Institute using the same primers. The raw sequences were assembled with SeqMan implemented in Lasergene v7.1 (DNASTAR, Madison, USA). The newly generated sequences in this study were submitted to GenBank.

### 2.3. Phylogenetic Analyses

Data on the generated sequences and the homologous sequences that were downloaded from GenBank were used to reconstruct the phylogenetic trees. Detailed sample information, including species names, voucher specimens, localities, GenBank accession numbers, and references, are listed in Table 1. The sequences of the three loci (LSU, *tef*1, and *rpb*2) were separately aligned using MAFFT [31] and examined in Bioedit v7.0.9 [32]. Missing sequences were coded as “N”, and the ambiguously aligned bases and introns of the protein-coding genes were retained in the final analyses. The final sequence alignments were deposited in TreeBase (ID 28589).

Phylogenetic analyses were performed following the methods that were described by Zhang et al. [42]. A maximum likelihood (ML) analysis was performed using RAxML v.7.2.6 [43] and Bayesian inference (BI) was performed using MrBayes 3.1.2 [44]. For both the ML and BI analyses, the most suitable substitution model for each gene partition was determined based on the Akaike Information Criterion (AIC) using MrModeltest v2.3 [45]. The default parameters were included for the ML analysis, except for selecting GTRGAMMAI as the model, and the statistical data were obtained by performing rapid non-parametric bootstrapping with 1000 replicates. A BI analysis using four chains was conducted using 30 million generations and the stoprul command with stopval set to 0.01. Bayesian trees were sampled every 100 generations, the first 25% of the generations were discarded as a burn-in, and the Bayesian posterior probabilities (BPP) were calculated from the posterior distribution of the retained Bayesian trees. The bootstrap support (BS) of ≥50% in the ML tree and BPP of ≥0.90 indicated statistical significance. The phylogenetic trees were visualised using FigTree v1.4.23.

## 3. Results

### 3.1. Molecular Phylogeny

**Figure 1:**
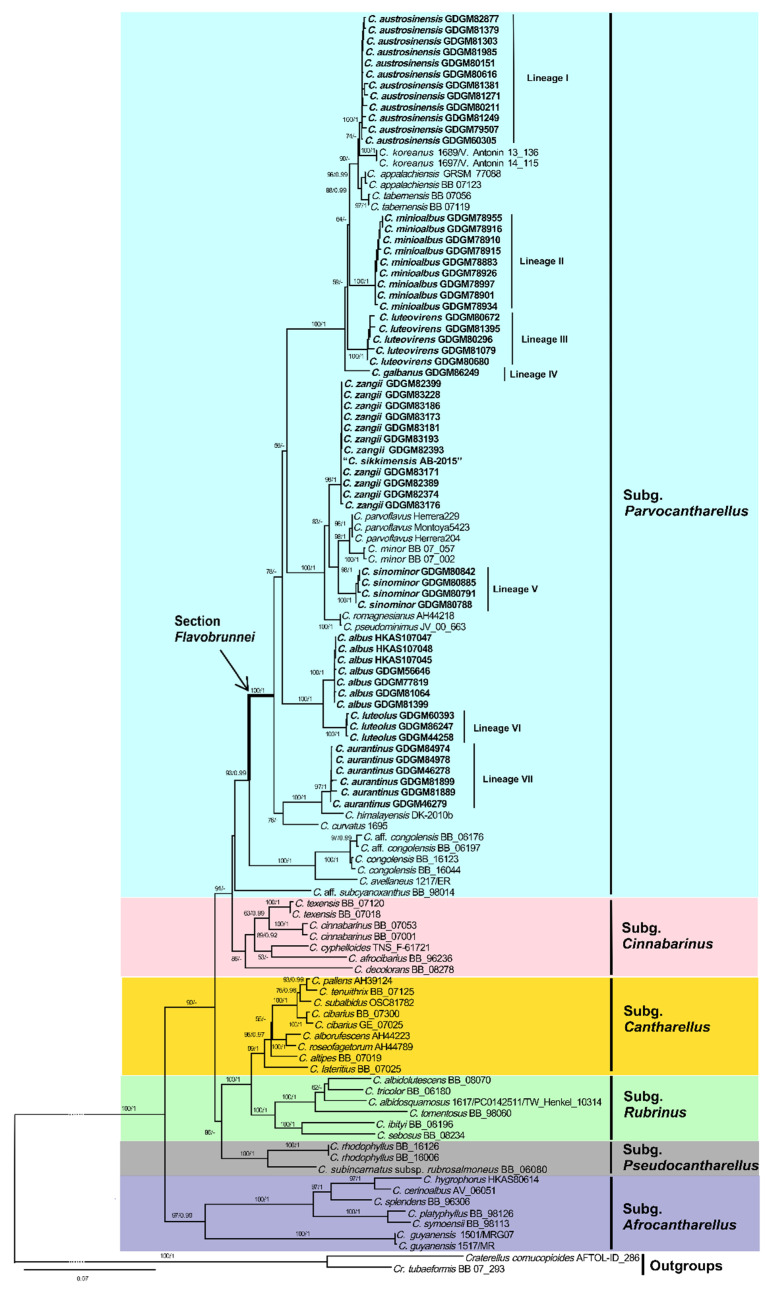
The phylogenetic tree of the representative species of *Cantharellus* that was inferred from a multigene (LSU + *tef*1 + *rpb*2) dataset by means of both ML and BI methods. *Craterellus cornucopioides* BB 07_293 and *Cr. tubaeformis* AFTOL ID_286 were used as outgroups. The maximum likelihood tree is depicted. The bootstrap supports (BS ≥ 50%) and Bayesian posterior probabilities (BPP ≥ 0.90) are shown on the supported branches. The species generated in this study are in black bold.

In the concatenated dataset (LSU + *tef*1 + *rpb*2), 304 sequences (108 for LSU, 101 for *tef*1, and 95 for *rpb*2) from 114 fungal collections were included. The alignment length was 3135 characters including gaps (1536 characters for LSU, 723 characters for *tef*1, and 876 characters for *rpb*2), of which 1847 characters were conserved, 226 were variable and parsimony-uninformative, and 1062 were parsimony-informative. *Craterellus cornucopioides* (L.) Pers. and *Cr. tubaeformis* were selected as outgroups based on recent studies [1,2,46]. The best models for the BI analysis of the concatenated dataset were GTR + I + G for LSU, K2P + I for *tef*1, and GTR + I for *rpb*2, respectively. The ML analysis resulted in a similar topology to the Bayesian analysis, and only the ML topology has been depicted in Figure 1.

Phylogenetic analyses that were based on the multi-locus dataset (LSU + *tef*1 + *rpb*2) showed that *C.* subg. *Parvocantharellus* formed a distinct clade in the genus *Cantharellus*, and seven new well-supported lineages were nested in this subgenus. Lineage I formed a well-supported terminal clade (100% BS and 1.00 BPP) and was closely related to *C. appalachiensis* R.H. Petersen and *C. tabernensis* Feib. & Cibula. Lineages II, III, and IV formed three isolated terminal branches with robust evidence (100% BS and 1.00 BPP). Lineage V formed sister relationships with *C. parvoflvus* M. Herrera, Bandala, & Montoya and *C. minor* Peck, and was also closely related to *C. romagnesianus* Eyssart. & Buyck. Lineage VI formed a sister relationship with *C. albus* S.P. Jian & B. Feng. Finally, Lineage VII was closely related to *C. himalayensis* D. Kumari, Ram. Upadhyay & Mod.S. Reddy and *C. curvatus* Buyck, R. Ryoo & Antonín. In addition, two known species, *C. albus* and *C. zangii*, that were originally reported from China, were strongly supported (100% BS and 1.00 BPP) in the phylogenetic trees, but a sequence named *C. sikkimensis* K. Das, Buyck, D. Chakr., Baghela, S.K. Singh & V. Hofst. was clustered with *C. zangii* in the multi-locus phylogenetic tree. 

**Figure 2:**
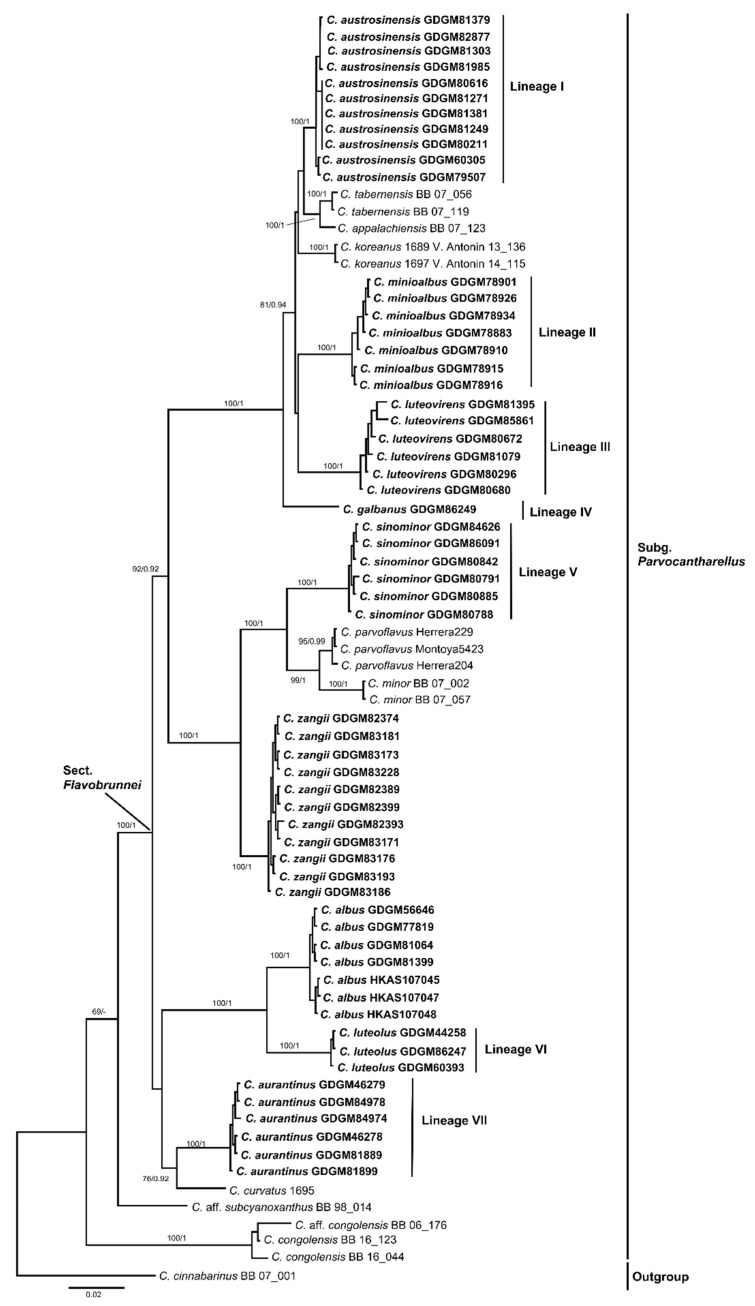
Phylogenetic tree of the representative species of the *Cantharellus* subgenus *Parvocantharellus* that was inferred from the *tef*1 dataset by means of both ML and BI methods. *Cantharellus cinnabarinus* BB 07_001 is used as the outgroup. The maximum likelihood tree is depicted. Bootstrap supports (BS ≥ 50%) and Bayesian posterior probabilities (BPP ≥ 0.90) are shown on the supported branches. The species generated in this study are in black bold.

In the *tef*1 dataset, 74 sequences from the 18 species were selected for the phylogenetic analyses. The length of the dataset was 706 characters including gaps, of which 448 characters were conserved, 22 were variable and parsimony-uninformative, and 236 were parsimony informative. *Cantharellus cinnabarinus* (Schwein.) Schwein. was selected as the outgroup based on the above multi-locus analyses. K2P + G4 was selected as the best model for BI. The ML and Bayesian analyses produced similar estimates of tree topologies, and only the tree that was inferred from the ML analysis is displayed (Figure 2). Species in the *C.* subg. *Parvocantharellus* formed three main subclades but without significant support. The seven new lineages were also strongly revealed in the phylogenetic tree and generated similar results with the multi-locus phylogenetic analysis. 

### 3.2. Taxonomy

**Figure 3:**
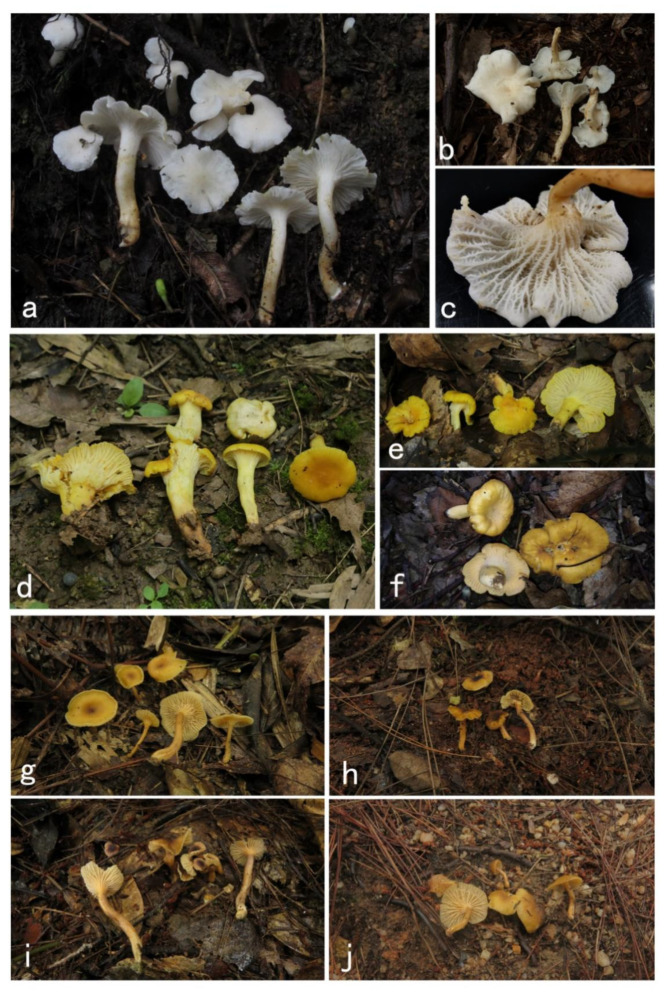
Species of *Cantharellus* subg. *Parvocantharellus* from China. (**a**–**c**) *C. albus* (**a**) GDGM56646; (**b**) GDGM73460; (**c**) GDGM81399; (**d**–**f**) *C. aurantinus* (**d**) GDGM46278 holotype (**e**) GDGM46279; (**f**) GDGM81889; (**g**–**j**) *C. austrosinensis* (**g**) GDGM81249 holotype (**h**) GDGM80151; (**i**) GDGM80211; (**j**) GDGM80296.

**Figure 4:**
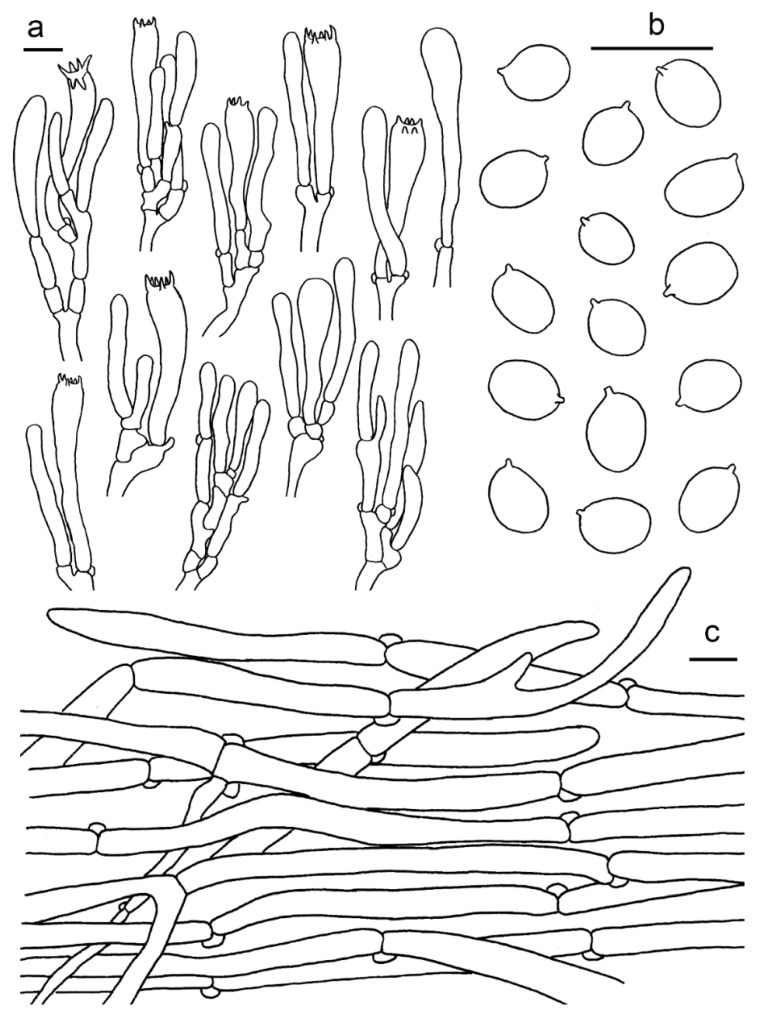
*Cantharellus albus* (GDGM56646). (**a**) Basidia, basidiola and elements of the subhymenium. (**b**) Basidiospores. (**c**) Pileipellis. Bars: (**a**,**b**) = 10 μm; (**c**) = 20 μm.

***Cantharellus albus*** in Jian, S.P.; Feng, B. Jian, Feng & Yang. *Phytotaxa* 2020, *470*, 137; Figure 3a–c and Figure 4.

Basidiomata small-sized. Pileus 18–42 mm broad, convex when young, then gradually to nearly applanate with a central shallow depression or broadly infundibuliform at maturity; surface dry, with appressed fibrillose squamules, white to yellowish white (4A1–4A2); margin wavy, incurved when young, decurved to slightly upturned at maturity, slightly changing to yellowish when handled, yellowish white to pale orange when dried (4A2–5A2, 4A3–5A3). Context white, 1–3 mm thick in the center of the pileus, sharply attenuate towards margin, unchanging or slightly changing to yellowish when cut. Hymenophore decurrent, close, poorly developed, composed of bifurcate and strongly interconnected low veins, usually less than 1 mm high, white to yellowish white, unchanging or slightly changing to yellowish when bruised. Stipe 20–60 × 2–8 mm, central, cylindrical or slightly tapering towards base, solid, smooth or with faintly scaly, concolourous with pileus, but in the lower part usually yellowish, slightly changing to yellowish when handled. All parts of basidioma becoming yellowish with 5% KOH solution. Odour milk fragrance, pleasant. Taste a little spicy. 

Basidiospores (100/4/4) 5.5–7.5 × (4–) 4.5–6 μm, L_m_ × W_m_ = 6.38 (±0.54) × 4.89 (±0.38) μm, Q = (1.1) 1.2–1.45 (1.62), Q_m_ = 1. 31 ± 0.11, broadly ellipsoid to subglobose, smooth, guttulate. Basidia 43–60 × 8–12 μm, 4–6-spored, narrowly clavate, colourless to hyaline in KOH; sterigmata 3–5 μm long. Pileipellis a cutis with long, repent and occasionally interwoven hyphae, subcylindrical cells that are 5–13 μm wide, thin-walled. Stipitipellis a cutis of cylindrical, parallel hyphae, 3–9 μm wide. Clamp connections abundant in all tissues.

Habitat and distribution—Growing in groups or gregariously under Fagaceae plants [*Castanopsis fissa* (Champ. ex Benth., and *Castanopsis* sp.) Rehd. et Wils.], mixed with other broadleaf trees in subtropical broadleaf forests. Known from southwest and southern China.

Specimen examined—CHINA. Guangdong Province, Guangzhou City, Baiyunshan, National Forest Park, alt. 160 m, 14 May 2016, Ming Zhang (GDGM45932); Same location, 15 June 2018, Ming Zhang (GDGM73460); Same location, 15 June 2019, Yong He (GDGM56646); Same location, 27 August 2019, Yong He (GDGM77819). 

Notes—*Cantharellus albus* was recently described from southwest China [2] and exhibits small white basidiomata and slightly changes to a yellowish colour when it is bruised or treated with 5% KOH solution. They have poorly-developed gill-like folds with strongly bifurcate and interconnected low veins, a distinct creamy aroma and a slightly spicy taste. They have a white-coloured basidiomata that changes to yellowish-white to pale orange colour when it is dried, and have broadly ellipsoid to subglobose basidiospores. The distinct morphological characteristics and the well-supported monophyletic lineage render it easily distinguished from other the *Cantharellus* species. In the present study, *C. albus* was redescribed based on the specimens that were from Guangdong province, which were compared to the description of *C. albus* in Jian et al. [2], and the macro- and micro-characteristics were almost identical. However, the size of the basidiospores in Jian’s specimens [6–8 × 5–7 μm, L_m_ × W_m_ = 6.9 (±0.48) × 5.92 (±0.62) μm] were larger than those in our specimens [5.5–7.5 × 4.5–6 μm, L_m_ × W_m_ = 6.38 (±0.54) × 4.89 (±0.38) μm]. The minor difference in the size of the basidiospores in Jian et al. [2] and the present study could be explained by small quantitative differences between the geographically distant populations or the number of measured basidiospores; this has often been noted in other *Cantharellus* species.

**Figure 5:**
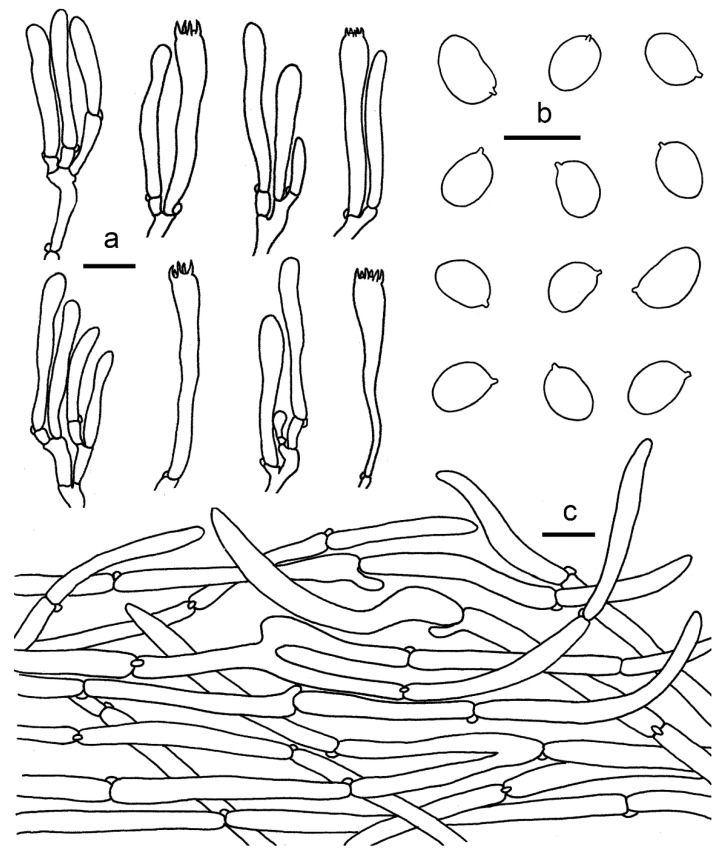
*Cantharellus aurantinus* (GDGM46278, Holotype!). (**a**) Basidia, basidiola and elements of the subhymenium. (**b**) Basidiospores. (**c**) Pileipellis. Bars: (**a**,**b**) = 10 μm; (**c**) = 20 μm.

***Cantharellus aurantinus*** Ming Zhang, Z.H. Zhang & T.H. Li sp. nov. Figure 3d–f and Figure 5.

MycoBank: MB840837.

Etymology—refers to the greyish-orange pileus colour.

Diagnosis—This species is characterized by its small basidiomata, light orange pileus, relatively well-developed hymenophore, and broadly ellipsoid basidiospores (6.5)7–9 × (4.5)5–6 μm in size. 

Type—CHINA. Henan Province, Xinyang City, Nanwan Lake Scenic Area, 420 m, N 32°11′, E 113°96′, on soil in *Castanopsis* spp. dominated forests, 18 July 2016, Ming Zhang (GDGM46278).

Basidiomata small-sized. Pileus 15–40 mm broad, convex when young, then gradually to nearly applanate with a central shallow depression at maturity; surface dry, smooth, light yellow, light orange, greyish yellow to greyish orange (2A5–6A5, 2B5–6B5), margin even, incurved when young, decurved to slightly upturned at maturity, unchanging when handled. Context white to yellowish white, 1.5–2.5 mm thick in the center of the pileus, sharply attenuate towards margin, unchanging when exposed. Hymenophore decurrent, relatively well developed, composed of bifurcate and interconnected low veins, in particular toward the cap margin, usually less than 1 mm high, pale yellow (2A3–4A3), unchanging when bruised. Stipe 20–40 × 8–12 mm, central, cylindrical or slightly tapering towards base, solid, smooth or with faintly scaly, pale yellow to pale orange (2A3–5A3), unchanging when handled. Odour not distinct.

Basidiospores (100/4/4) (6.5–)7–9 × (4.5–)5–6 μm, L_m_ × W_m_ = 7.95(±0.57) × 5.51(±0.42) μm, Q = (1.16)1.3–1.6(1.7), Q_m_ = 1.45±0.13, broadly ellipsoid, smooth, guttulate. Basidia 48–70 × 8–10 μm, 4–6-spored, narrowly clavate, colourless to hyaline in KOH; sterigmata 3–8 μm long. Pileipellis a cutis with long, repent and occasionally interwoven hyphae, subcylindrical cells that are 5–10 μm wide, thin-walled, obtusely rounded at the top. Stipitipellis a cutis of cylindrical, parallel hyphae, 3–12 μm wide. Clamp connections abundant in all tissues. 

Habitat and distribution—Growing in solitary or scattered under Fagaceae trees that are mixed with other broadleaf trees in subtropical broadleaf forests. Known from Henan and Jiangsu Province, China.

Specimen examined—CHINA. Henan Province, Xiyang City, Nanwan Lake Scenic Area, alt. 420 m, on soil under broadleaf forests, 18 July 2016, Ming Zhang (GDGM46279, GDGM46413). Jiangsu Province, Nangjing City, Tzu-chin Mountain Scenic Area, alt. 300 m, 5 September 2020, Zi-Han Zhang (GDGM81888, GDGM81889, GDGM81899); Same location, 24 May 2021, Zi-Han Zhang (GDGM84972); 5 June 2021, Zi-Han Zhang (GDGM84974, GDGM84975, GDGM84978).

Notes—The distinctive morphological features of *C. aurantinus* are the light orange to greyish-orange pileus, the pale yellow, gill-like folds with bifurcate and interconnected low veins, the broadly ellipsoid basidiospore, and the thin-walled hyphae of the pileipellis. The phylogenetic analyses supported *C. aurantinus* as an isolated lineage (Lineage VII) that is closely related to *C. curvatus* and *C. himalayensis*. However, *C. curvatus*, recently reported from South Korea, differs by its small and slender basidiomata, dull yellow to orangish-yellow pileus, and shorter basidia (42–55 × 9.5–12 µm) [33]. *Cantharellus himalayensis*, that is reported from India, differs by its large basidiomata, yellowish pileus with pecan-brown scales at the center, relatively small basidiospores (6–8 × 4.5–6 µm), and partially gelatinous pileipellis [38].

In the field, *C. aurantinus* is easily misidentified as *C. cibarius*, as both species share a yellow-orange pileus. However, *C. cibarius* belongs to the subg. *Cantharellus*, and differs by its relatively large basidiomata, well-developed hymenophore of up to 3 mm in depth, longer basidia (80–105 × 7–9 µm) and thick-walled pileipellis hyphae [9,18]. 

**Figure 6:**
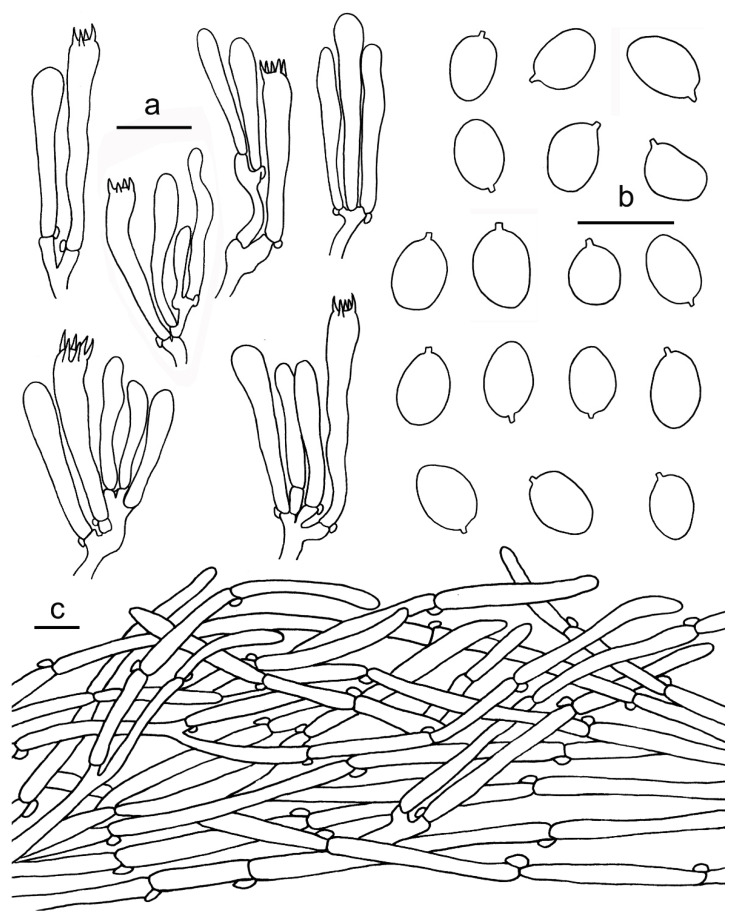
*Cantharellus austrosinensis* (GDGM81249, Holotype!). (**a**) Basidia, basidiola and elements of the subhymenium. (**b**) Basidiospores. (**c**) Pileipellis. Bars: (**a**,**b**) = 10 μm; (**c**) = 20 μm.

***Cantharellus austrosinensis*** Ming Zhang, C.Q. Wang & T.H. Li sp. nov. Figure 3g–j and Figure 6.

MycoBank: MB840652.

Etymology—refers to the distribution of this species in southern China.

Diagnosis—This species is characterized by its small basidiomata, pastel yellow to greyish-yellow pileus with a greyish-orange to brownish-orange center, pale yellow to light yellow hymenophore that is composed of bifurcate and interconnected low veins, elliptical to broadly elliptical basidiospores 6–8 × 4.8–6 μm, and the thin-walled hyphae of the pileipellis.

Type—CHINA. Guangdong Province, Shaoguan City, Renhua County, Danxiashan National Nature Reserve, alt. 199 m, on soil under *Pinus massoniana*, 4 June 2020, Ming Zhang (GDGM81249).

Basidiomata small-sized. Pileus 12–30 mm broad, applanate with center depressed, not perforate, margin incurved when young, applanate or slightly reflexed with age, obscure striated on surface; subfleshy to slightly membranous; surface dry, glabrous or tomentosus at central, pastel yellow, light yellow to greyish yellow at mass (3A5–5A5, 3B5–4B5), with a greyish orange to brownish orange center (5B5–6B5, 5C5–6C5), often with reddish brown tinge in some specimens (8D7–9D7). Context thin, 0.5–1.5 mm thick in the center of pileus, fibrous, pale yellow to light yellow (3A3–3A5), unchanging when bruised. Hymenophore decurrent, but with a clearly delimitation from the stipe surface, gill-like, well or poorly developed, ridges 1–2 mm high, composed of bifurcate and interconnected low veins, pale yellow to light yellow (3A3–4A3, 3A5–4A5), unchanging when bruised. Stipe 10–40 mm long, 2–5 mm thick, subcylindrical, enlarged downward, smooth or with faintly scaly, hollow, concolourous with pileus, darker and more somber than lamellae. Odour not special. Taste mild.

Basidiospores (100/4/4) 6–8 × 4.8–6 μm, L_m_ × W_m_ = 7.05(±0.51) × 5.192(±0.34) μm, Q = (1.08)1.2–1.45(1.6), Q_m_ = 1.36 ± 0.097, elliptical to broadly elliptical. Basidia 50–55 × 7–9 μm, clavate, with 5–6(–7) sterigmata. Pileipellis a cutis, composed of interwoven hyphae 5–12 μm in diam., colourless, thin-walled. Hymenophoral trama composed of cylindrical hyphae 7–10 μm in diam. Stipitipellis a cutis of cylindrical, parallel hyphae, 4–12 μm wide, branched, septate. Clamp connections abundant in all tissues.

Habitat and distribution—Solitary or scattered under *Pinus massoniana* Lamb. mixed with other broadleaf trees. Known from southern China.

Specimen examined—CHINA. Guangdong Province, Shaoguan City, Renhua County, Danxiashan National Natural Reserve, alt. 103 m, 1 May 2020, Ming Zhang (GDGM79507); same location, alt. 92 m, 5 June 2020, Li-Qiang Wu (GDGM81247); Feihuashui, alt. 152 m, 3 June 2020, Ming Zhang (GDGM81616); Guanyinshan, alt. 187 m, 27 May 2020, Ming Zhang (GDGM80151); Ruyuan County, Nanling National Natural Reserve, alt. 550 m, 10 June 2020, Ming Zhang (GDGM80211). 

Notes—The presence of small basidiomata, a pastel yellow to greyish-yellow pileus with a greyish-orange to brownish-orange centre, thin-walled hyphae, and abundant clamp connections enable the classification and placement of *C. austrosinensis* in the subg. *Parvocantharellus*. In the phylogenetic trees, the new species was closely related to *C. appalachiensis*, *C. koreanus* Buyck, Antonín & Ryoo, and *C. tabernensis*. However, *C. appalachiensis* and *C. tabernensis*, that are both described from North America, can be distinguished by their relatively large and more robust basidiomata, with a pileus that is usually up to 50 mm in width. Additionally, *C. appalachiensis* differs in the existence of its drab yellow to dull brown pileus, its relatively large and narrow basidiospores (6.6–8.9 × 4.4–5.9 µm or 6–10.5 × 4–6 µm), and its association with oaks and other hardwoods [47,48]. *Cantharellus tabernensis* also differs in the presence of its dull orange-yellow to yellowish-brown pileus, vivid orange-yellow hymenophore, its well-developed gills that are up to 3 mm in depth, vivid orange-yellow stipe that is up to 8 mm in diameter, narrow basidiospores with a large Q value (1.49–1.52), and narrow hymenophoral trama hyphae (3–6 µm in diameter) [49]. *Cantharellus koreanus*, originally described from the temperate region of the Republic of Korea, differs in the presence of its dirty yellow-brown to pale brown pileus with a brown to dark brown centre, yellow to greyish stipe, relatively narrow basidiospores (4.2–5.5 μm in breadth), and its association with various deciduous trees (*Carpinus laxiflora*, *Castanea crenata*, and *Quercus mongolica*) mixed with coniferous trees (*Pinus densiflora*) [16]. In contrast, *C. austrosinensis* is distributed in subtropical regions of China and is currently only known to be associated with *Pinus massoniana*. 

*Cantharellus quercophilus* Buyck, D.P. Lewis, Eyssart. & V. Hofst., belonging to the subg. *Cantharellus*, resembles a *C. austrosinensis* with small basidiomata, however, *C. quercophilus*, that was originally reported in the USA, differs in the presence of its pale brown to greyish-yellow pileus, cream to pale yellowish hymenophore that is sparsely forked, its greyish-buff with a lilac tinged context that changes to a yellow to reddish-brown colour when it is bruised, and its association with *Quercus stellata* [50]. 

**Figure 7:**
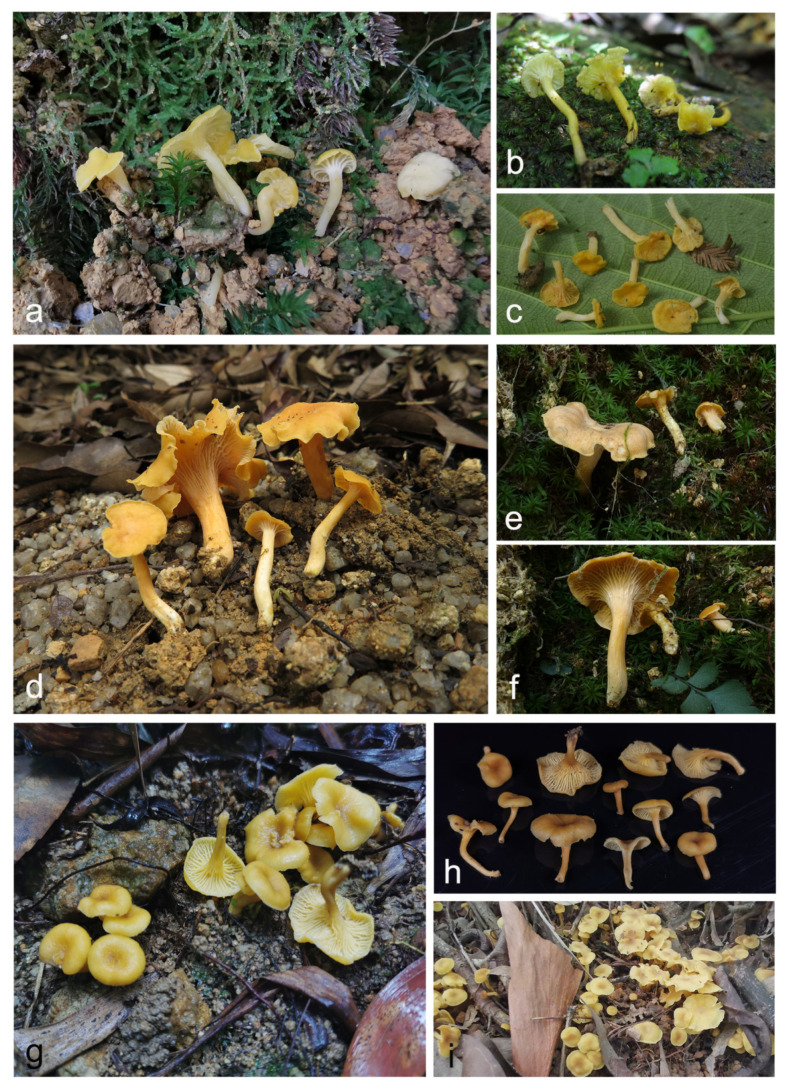
Species of *Cantharellus* subg. *Parvocantharellus* from China. (**a**–**c**) *C. galbanus* (**a**) GDGM86429 holotype! (**b**) GDGM43100; (**c**) GDGM60568); (**d**–**f**) *C. luteolus* (**d**) GDGM60393 holotype! (**e**,**f**) GDGM86247); (**g–i**) *C. luteovirens* (**g**,**h**) GDGM80672 holotype! (**i**) GDGM81079.

**Figure 8:**
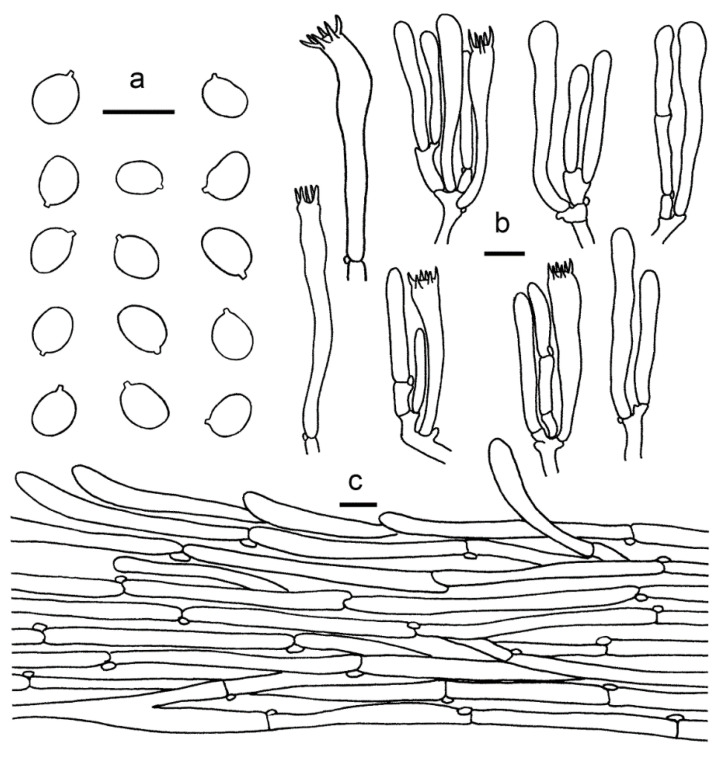
*Cantharellusgalbanus* (GDGM86249, Holotype!). (**a**) Basidiospores. (**b**) Basidia, basidiola and elements of the subhymenium. (**c**) Pileipellis. Bars: (**a**,**b**) = 10 μm; (**c**) = 20 μm.

***Cantharellus galbanus*** Ming Zhang, C.Q. Wang & T.H. Li sp. nov. Figure 7a–c and Figure 8.

MycoBank: MB840835.

Etymology—refers to the greenish-yellow basidiomata.

Diagnosis—This species is characterized by its small basidiomata, greenish-yellow to yellow pileus, well-developed gill-like ridges that are usually forked at the margin, relatively small basidiospores 6–7.5 × 4.8–5.5 μm, and thin-walled hyphae of the pileipellis.

Type—CHINA. Hainan Province, Ledong County, Jianfengling National Nature Reserve, alt. 950 m, on soil under Fagaceae trees mixed with other broadleaf trees in tropical broadleaf forests, 13 July 2021, Ming Zhang (GDGM86249).

Basidiomata small-sized. Pileus 5–10 mm broad, convex when young, then gradually to nearly applanate with a central shallow depression or finally broadly infundibuliform at maturity; surface dry, glabrous to subtomentosus, hygrophanous when wet, greenish yellow (1A6), light yellow (1A5–3A5), yellow (2A6–2B6) to greyish yellow (2B5–3C5), margin incurved and irregularly wavy. Context less than 1 mm thick, yellowish, unchanging when bruised. Hymenophore distant, well developed, composed of decurrent and usually forked gill-folds, less than 1 mm depth, yellowish white to pale yellow (1A2–2A2, 1A3–2A3). Stipe 10–15 × 1–2 mm, cylindrical, or gradually slender towards base, central, hollow, surface smooth, slightly waxy, concolourous with pileus or paler to pastel yellow (1A4–2A4), greenish yellow to yellowish grey (1B2–2B2). Taste mild. Odour fruity.

Basidiospores (50/2/2) 6–7.5 × 4.8–5.5 μm, L_m_ × W_m_ = 6.77(±0.36) × 5.19(±0.32) μm, Q = (1.18)1.27–1.4, Q_m_ = 1.31±0.07, elliptical to broadly elliptical, uniguttulate to multiguttulate, smooth, hyaline, inamyloid, with refringent contents. Basidia 52–76 × 7–8 μm, 4–6-spored, narrowly clavate with large number of vacuoles, sterigmata 4–6 μm long; basidiole 50–80 × 7–10 μm, numerous, clavate. Hymenial cystidia absent. Hymenophoral trama filamentous, composed of colourless and branched hyphae, hyphae up to 13 μm in diam., septate, thin-walled. Pileipellis a cutis, composed of horizontal to ascending, subparallel, cylindrical and branched, thin-walled hyphae arranged mostly in irregular patern; septa clamped; terminal cells 5–17 μm wide, mostly cylindrical to subclavate, slightly appendiculate. Stipitipellis a cutis of cylindrical, parallel hyphae, 5–13 μm wide, branched, septate, mostly cylindric with clavate to subfusoid. Stipe trama with hyphae 3–15 μm wide, clamped, septate. Clamp connections abundant in all tissues.

Habitat and distribution—Growing in groups or gregariously under Fagaceae trees mixed with other broadleaf trees in tropical broadleaf forests. Known from southern China. 

Specimen examined—CHINA. Hainan Province, Changjiang County, Bawangling National Natural Reserve, 942 m, 7 July 2013, Ming Zhang (GDGM43100); Hainan Province, Ledong County, Jianfengling National Natural Reserve, 950 m, 17 June 2017, Ming Zhang (GDGM60568).

Notes—*Cantharellus galbanus* is characterized by the presence of its small basidiomata, greenish-yellow pileus, distant and well-developed hymenophore, and relatively small basidiospores (6–7.5 × 4.8–5.5 μm). The molecular phylogenetic analysis that was based on a single specimen showed that *C. galbanus* formed an independent clade (Lineage IV) and was clearly distinguished from the other species in the subg. *Parvocantharellus*, fully supporting the identification of *C. galbanus* as a distinct species. *Cantharellus citrinus* Buyck, R. Ryoo & Antonín, recently reported from South Korea, is morphologically similar to *C. galbanus*, as both species share small, lemon-yellow basidiomata. However, *C. citrinus* belongs to the subg. *Cinnabarini* and differs in its relatively poorly-developed hymenophore with transversely irregular anastomosis, and large basidiospores (7.6–8.4 × 5.4–5.9 μm) [33]. 

**Figure 9:**
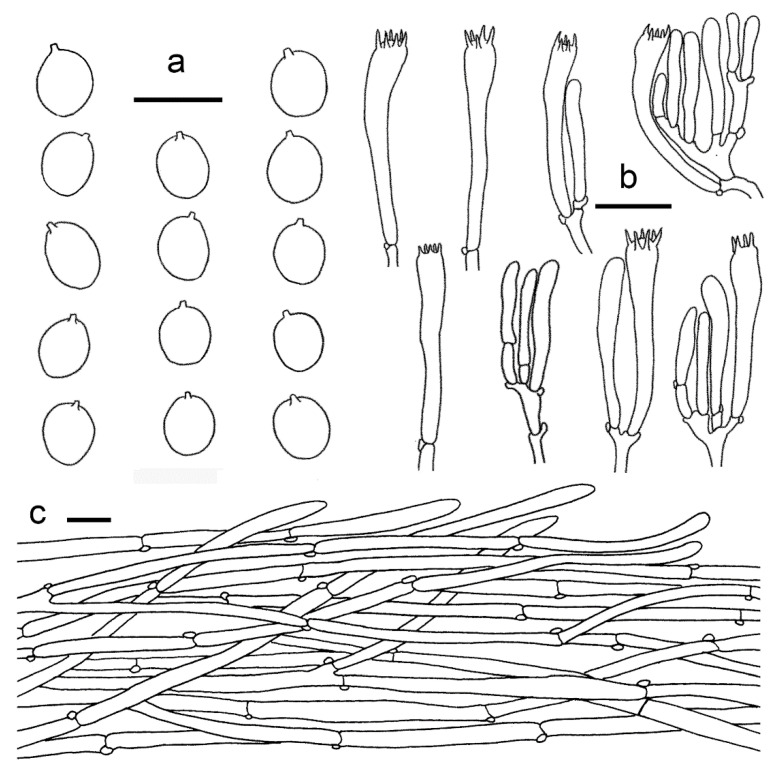
*Cantharellus luteolus* (GDGM60393, Holotype!). (**a**) Basidiospores. (**b**) Basidia, basidiola and elements of the subhymenium. (**c**) Pileipellis. Bars: (**a**) = 10 μm; (**b**,**c**) = 20 μm.

***Cantharellus luteolus*** Ming Zhang, C.Q. Wang & T.H. Li sp. nov. Figure 7d–f and Figure 9.

MycoBank: MB840836.

Etymology—refers to the yellowish-orange pileus colour.

Diagnosis—This species is characterized by its small basidiomata, yellow to orange pileus, well-developed gill-like ridges mostly forked at the margin, elliptical to subglobose basidiospores, and thin-walled hyphae of pileipellis.

Type—CHINA. Hainan Province, Ledong County, Jiangfengling National Nature Reserve, alt. 950 m, on soil under Fagaceae trees that are mixed with other broadleaf trees in tropical broadleaf forests, 17 June 2017, Ming Zhang (GDGM60393).

Basidiomata small-sized. Pileus 20–32 mm broad, convex when young, then applanate with center depressed at mature, surface dry, tomentosus, pastel yellow, yellow, light yellow, yellowish orange, greyish orange, light orange to orange (3A4–5A4, 3A6–6A6, 3B4–5B4), margin incurved and irregularly wavy. Hymenophore well developed, composed of decurrent, mostly forked and strongly interveined gill-folds, less than 1 mm depth, light yellow (3A5–4A5), greyish yellow to greyish orange (3B5–5B5). Stipe 20–30 × 3–7 mm, cylindrical, or gradually slender towards base, central, hollow, surface smooth, slightly waxy, concolourous with pileus or paler to light yellow, greyish yellow (3A5–4B5) to light orange (4A5). Context white to yellowish, 1–2 mm thick, unchanging when bruised. Taste mild. Odour fruity.

Basidiospores (75/3/3) 7–8 × 5.2–6.5 μm, L_m_ × W_m_ = 7.44(±0.46) × 6.04(±0.29) μm, Q = (1.12)1.16–1.33(1.36), Q_m_ = 1.23 ± 0.07, elliptical to subglobose, uniguttulate to multiguttulate, smooth, hyaline, inamyloid, with refringent contents. Basidia 60–85 × 7–10 μm, 4–6-spored, narrowly clavate with large number of vacuoles, sterigmata 5–10 μm long; basidiole 51–85 × 6–10 μm, numerous, clavate. Hymenial cystidia absent. Hymenophoral trama filamentous; hyphae 3–9 μm diam., branched, septate. Pileipellis a cutis, composed of horizontal to ascending, subparallel, cylindrical and branched, thin-walled hyphae arranged mostly in irregular patern; septa clamped; terminal cells mostly cylindrical to subclavate, up to 150 μm long, slightly appendiculate. Stipitipellis a cutis of cylindrical, parallel hyphae, 3–12 μm wide, branched, septate, mostly cylindric with clavate to subfusoid. Stipe trama with hyphae 3–9 μm wide, clamped, septate. Clamp connections abundant in all tissues.

Habitat and distribution—Growing solitary or scattered under Fagaceae trees that are mixed with other broadleaf trees in tropical broadleaf forests. Known from southern China. 

Specimen examined—CHINA. Hainan Province, Changjiang County, Bawangling National Natural Reserve, 942 m, 6 July 2013, Ming Zhang (GDGM44258); Hainan Province, Ledong County, Jianfengling National Natural Reserve, 942 m, 13 July 2021, Ting Li (GDGM86247).

Notes—The following combination of characteristics that included small basidiomata, yellow to orange pileus, greyish-yellow to greyish-orange hymenophores, elliptical to subglobose basidiospores, and thin-walled hyphae of the pileipellis, made *C. luteolus* easily distinguishable from the other species in *Cantharellus*. Genetically, *C. luteolus* is a monophyletic taxon (Lineage VI) that is significantly related to the Chinese species *C. albus*, together forming a significantly monophyletic clade. However, *C. albus* can be easily distinguished by the presence of its robust, white basidiomata that slightly changes to a yellowish colour when it is bruised, with a strongly bifurcate and interconnected hymenophore, and relatively small basidiospores [2], above study in *C. albus*.

**Figure 10:**
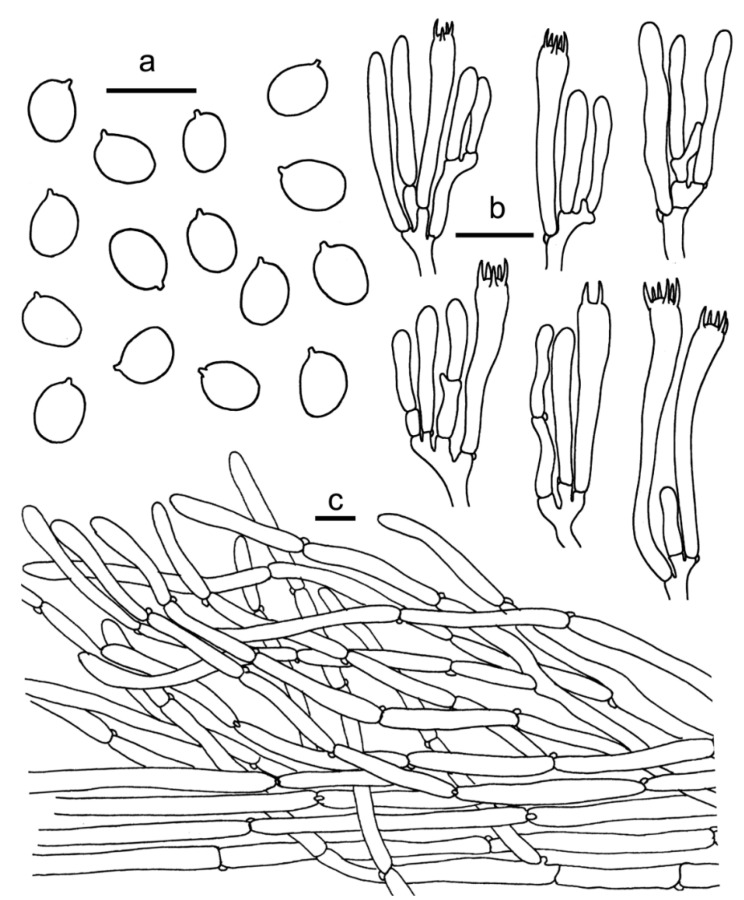
*Cantharellus luteovirens* (GDGM80672 Holotype!). (**a**) Basidiospores. (**b**) Basidia, basidiola and elements of the subhymenium. (**c**) Pileipellis. Bars: (**a**,**b**) = 10 μm; (**c**) = 20 μm.

***Cantharellus luteovirens*** Ming Zhang, C.Q. Wang & T.H. Li sp. nov. Figure 7g–i and Figure 10.

MycoBank: MB840653.

Etymology—refers to the yellowish-orange basidiomata.

Diagnosis—This species is characterized by its small, yellow to yellowish-orange basidiomata, hygrophanous pileus surface, poorly-developed hymenophore that is composed of strongly bifurcate and interconnected low veins, and broadly ellipsoid basidiospores that measure 6–7(–7.5) × (4.5–)4.8–5.5(–6) μm.

Type—CHINA. Guangdong Province, Guangzhou City, Baiyun Mountain, alt. 130 m, 16 June 2020, Ming Zhang (GDGM80672).

Basidiomata small-sized. Pileus 15–42 mm broad, convex when young, then gradually to nearly applanate with a central shallow depression or broadly infundibuliform at maturity; surface dry to hygrophanous, smooth, pastel yellow (2A4–4A4), light yellow (2A5–4A5), yellow to yellowish orange (3A6–4A6), usually with greyish yellow (3C5–4C5) tinct at center, margin wavy, incurved when young, decurved to slightly upturned at maturity, unchanging when handled. Context yellowish white, 1–1.3 mm thick in the center of the pileus, sharply attenuate towards margin, unchanging when exposed. Hymenophore decurrent, subdistant to close, poorly developed, composed of bifurcate and strongly interconnected low veins, usually less than 1 mm high, yellowish white to pale yellow (2A2–3A2, 2A3–3A3), unchanging when bruised. Stipe 20–30 × 2–3 mm, central, cylindrical or slightly tapering towards base, hollow, smooth, concolourous or paler than pileus, unchanging when handled. Odour none, taste mild.

Basidiospores (100/4/4) 6.0–7.0(7.5) × (4.5)4.8–5.5(6) μm, L_m_ × W_m_ = 6.79(±0.40) × 5.07(±0.30) μm, Q = (1.15)1.27–1.5(1.55), Qm = 1.34±0.093, broadly ellipsoid to subglobose, smooth, guttulate. Basidia 40–70 × 6–10 μm, 2–6-spored, narrowly clavate, colourless to hyaline in KOH; sterigmata 3–7 μm long. Pileipellis a cutis, composed of repent and occasionally branched hyphae, subcylindrical cells that are 6–13 μm wide, up to 200 μm long, thin-walled. Stipitipellis a cutis of cylindrical, parallel hyphae, 4–8 μm wide, occasionally up to 12 μm wide. Clamp connections abundant in all tissues.

Habitat and distribution—Growing in groups or gregariously under *Acacia auriculiformis* A. Cunn. ex Benth and *Acacia mangium* mixed other broadleaf trees in subtropical forests. Currently only known from Guangdong Province in southern China.

Specimen examined—CHINA. Guangdong Province, Guangzhou City, Baiyun Mountain, alt. 100 m, 8 May 2020, Xi-Shen Liang (GDGM81079); Same location, alt. 136 m, 14 May 2016, Ming Zhang (GDGM45899); Same location, alt. 218 m, 28 May 2020, Ming Zhang (GDGM81395); Same location, alt. 183 m, 12 June 2020, Jun-Yan Xu (GDGM80296).

Notes—*Cantharellus luteovirens* is characterised by the presence of its small pastel yellow to yellowish-orange basidiomata, poorly-developed hymenophore with bifurcate and strongly interconnected low veins, broadly ellipsoid to subglobose basidiospores, and thin-walled pileipellis hyphae. These traits enable the classification and placement of *C. luteovirens* into the subg. *Parvocantharellus*. The molecular phylogenetic analyses showed that all of the *C. luteovirens* specimens formed a distinct lineage (Lineage III) close to *C. minioalbus* (Lineage II). However, *C. luteovirens* is morphologically different from *C. minioalbus* by its pastel yellow to yellowish-orange basidiomata, poorly-developed hymenophore, and relatively large basidiospores.

*Cantharellus galbanus* is extremely morphologically similar to *C. luteovirens*. However, *C. galbanus* differs by its small basidiomata, greenish pileus colour, relatively distant and well-developed hymenophore, and tropical distribution. In the phylogenetic trees, the two species formed two distinct monophyletic taxa and could easily be separated from each other owing to their branch lengths. 

Additionally, *C. koreanus*, *C. minor*, and *C. tabernensis* are also morphologically similar to *C. luteovirens*, owing to the presence of small basidiomata and a yellowish to orange tinct pileus. However, the former three species can be distinguished from *C. luteovirens* in the field by their relatively distant and well-developed hymenophores. Additionally, *C. koreanus* differs by the presence of its dirty yellowish-brownish to pale brown pileus, with a brown centre, and relatively large but narrow basidiospores [6–8(–9) × 4.2–5.5(–6.5) μm] [16]. *Cantharellus minor* differs by the presence of its egg-yellow to orange pileus, long stipe, and large basidiospores 8–11 × 5–7 μm [51,52,53]. *Cantharellus tabernensis* differs by the presence of its dull orange-yellow to yellowish-brown pileus, vivid orange-yellow hymenophore and stipe, relatively large basidiospores (6–9 × 4–6 μm), and small basidia (35–55 × 5–8 μm) [49]. 

**Figure 11:**
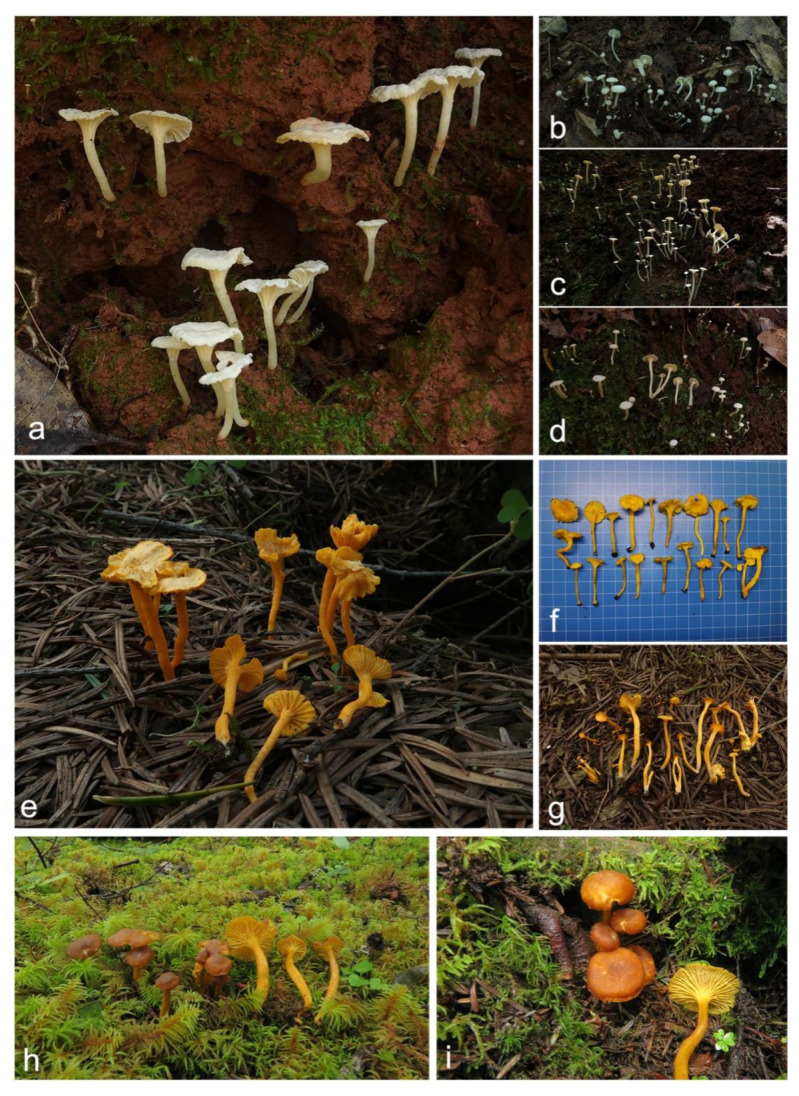
Species of *Cantharellus* subg. *Parvocantharellus* from China. (**a**–**d**) *C. minioalbus* (**a**) GDGM78926; (**b**) GDGM78901 holotype! (**c**) GDGM78934; (**d**) GDGM78910; (**e**–**g**) *C. sinominor* (**e**) GDGM80842 holotype (**f**) GDGM80788; (**g**) GDGM80885; (**h**–**i**) *C. zangii* (**h**) GDGM83181; (**i**) GDGM83193.

**Figure 12:**
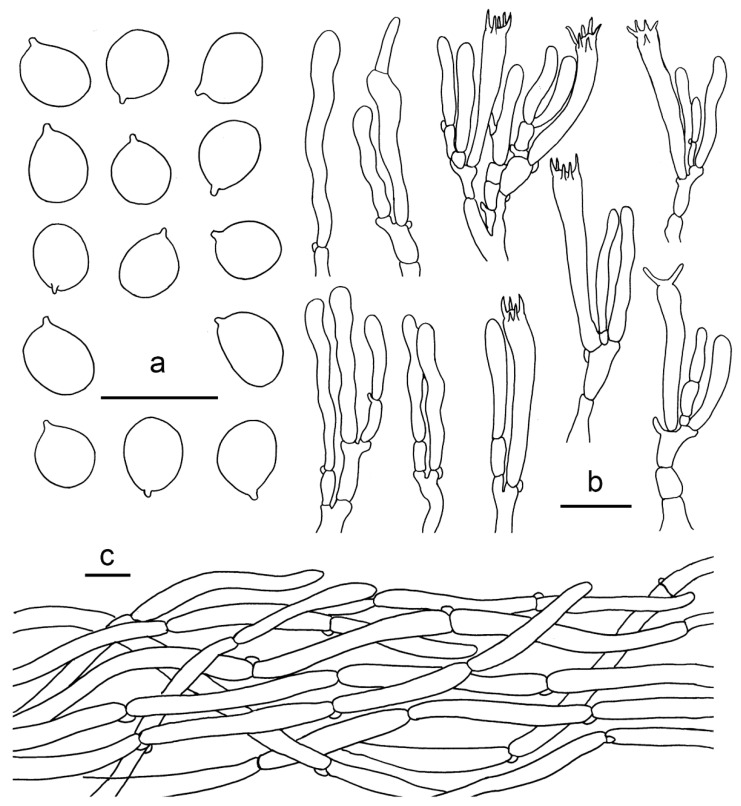
*Cantharellus minioalbus* (GDGM78901, Holotype!). (**a**) Basidiospores. (**b**) Basidia, basidiola and elements of the subhymenium. (**c**) Pileipellis. Bars: (**a**,**b**) = 10 μm; (**c**) = 20 μm.

***Cantharellus minioalbus*** Ming Zhang, C.Q. Wang & T.H. Li sp. nov. Figure 11a–d and Figure 12.

MycoBank: MB840654.

Etymology—refers to the small white basidiomata.

Diagnosis—This species can be easily distinguished from others in *Cantharellus* by its small and white-coloured basidiomata, gill-like hymenophore that is well-developed with bifurcate and interconnected low veins, and growing in groups or gregariously under broadleaf trees.

Type—CHINA. Yunnan Province, Puer City, Taiyanghe National Forest Park, alt. 1616 m, N 22°36′24.8″, E 101°05′21.6″, 24 September 2019, Ming Zhang (GDGM78901).

Basidiomata small-sized. Pileus 3–10 mm broad, convex when young, then gradually to nearly applanate with a central shallow depression or finally broadly infundibuliform at maturity; surface dry, with appressed fibrillose scales, white to yellowish white, margin wavy, incurved when young, decurved to slightly upturned with maturity, unchanging when handled. Context yellowish white, unchanging when exposed. Hymenophore decurrent, but with a clearly delimitation from the stipe surface, distant, with well-defined gill-like folds, relatively well developed, frequently forking towards pileus margin, with lower irregular anastomosis amongst the folds, white to pale yellow, unchanging when bruised. Stipe 15–30 × 1.5–2.5 mm, central, cylindrical or slightly inflated towards base, solid, smooth or faintly scaly, concolourous with pileus, unchanging when handled. Odour not distinct.

Basidiospores (100/4/4) 4.5–7 × 4–5.5(–6.2) μm, L_m_ × W_m_ = 5.71(±0.64) × 4.87(±0.49) μm, Q = (1)1.1–1.27(1.37), Q_m_ = 1.17 ± 0.07, broadly ellipsoid to subglobose, smooth, guttulate. Basidia 44–66 × 6–8 μm, 2–6-spored, narrowly clavate, colourless to hyaline in KOH, with 2–6 sterigmata; sterigmata 3–7.5 μm long. Hymenophoral trama irregular, composed of colourless and branched hyphae, 3–20 μm wide, septate, thin-walled. Pileipellis a cutis with long, repent, branched, and usually interwoven hyphae, subcylindrical cells that are 3–13 μm wide, thin-walled; terminal cells appressed to suberect, mostly cylindrical, up to 200 μm long and 5‒13 μm wide. Stipitipellis a cutis of cylindrical, parallel hyphae, 3–7 μm wide, terminal cells clavate or cylindrical, 5–10 μm wide. Clamp connections abundant in all tissues.

Habitat and distribution—Growing in groups or gregariously under *Castanopsis* sp. and *Fagus* sp. in tropical broadleaf forests. Currently only known from Yunnan Province, Southwest China.

Specimen examined—CHINA. Yunnan Province, Puer City, Simao District, Taiyanghe National Forest Park, alt. 1616 m, 24 September 2019, Ming Zhang (GDGM78915, GDGM78916, GDGM78926, GDGM78934), Jun-Yan Xu (GDGM78901); Same locality, alt. 1662 m, 25 September 2019, Ming Zhang (GDGM78955), Jun-Yan Xu (GDGM78997).

Notes—*Cantharellus minioalbus* is characterised by the presence of its small white basidiomata, broadly infundibuliform pileus that is covered with fibrillose scales, distant and well-defined gill-like hymenophore that are frequently forking towards the pileus margin, with the existence of few abnormal anastomosis among the folds, broadly ellipsoid to subglobose basidiospores, and thin-walled hyphae of pileipellis. These traits enable the classification and placement of *C. minioalbus* into the subg. *Parvocantharellus*. The molecular phylogenetic analyses showed that the new species formed an isolated lineage in the subg. *Parvocantharellus* and was genetically distinct from all of the other *Cantharellus* taxa with sequence data.

*Cantharellus albus* is similar to *C. minioalbus*, as both species share white basidiomata. However, *C. albus*, redescribed above, differs by the alteration of the basidiomata colour from white to yellow when it is bruised, and by the presence of a poorly-developed hymenophore with variously forked or strongly anastomosing veins, and relatively large basidiospores [5.5–7.5 × (4–) 4.5–6 μm].

**Figure 13:**
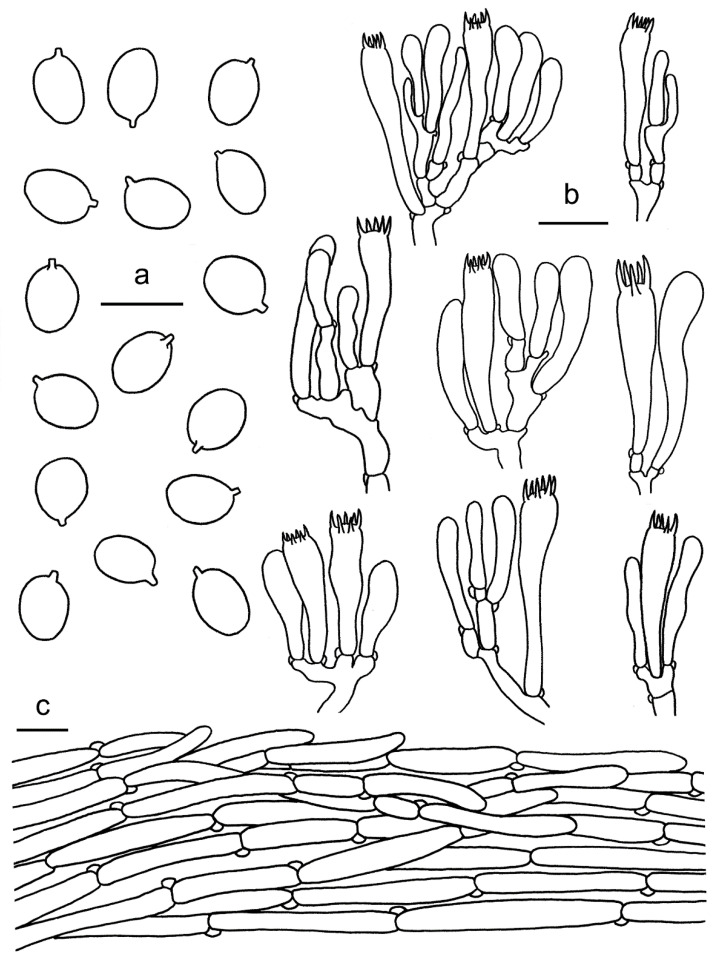
*Cantharellus sinominor* (GDGM80842, Holotype!). (**a**) Basidiospores. (**b**) Basidia, basidiola and elements of the subhymenium. (**c**) Pileipellis. Bars: (**a**) = 10 μm; (**b**–**c**) = 20 μm.

***Cantharellus sinominor*** Ming Zhang, C.Q. Wang & T.H. Li sp. nov. Figure 11e–g and Figure 13.

MycoBank: MB840655.

Etymology—refers to the species described from China and is similar to *C. minor*.

Diagnosis—This species is characterized by its small and light yellow basidiomata with a relatively longer stipe, the stipe is usually longer than the diameter of the pileus. It has well-developed gill-like ridges that are mostly forked at the margin, as well as elliptical to elongate elliptical basidiospores, and thin-walled hyphae of the pileipellis.

Type—CHINA. Guizhou Province, Longli Town, Longjiashan Forest Park, alt. 1000 m, on soil under *Keteleeria* sp. and *Picea* sp. dominated forests, 5 July 2020, Ming Zhang (GDGM80842).

Basidiomata small-sized. Pileus 10–23 mm broad, applanate with center depressed, not perforate, margin incurved when young, applanate or slightly reflexed with age, obscure striate on surface; subfleshy to slightly membranous; surface dry, subtomentosus, greyish yellow to greyish orange at central (3B4–5B4), gradually fading to light yellow to light orange toward margin (3A4–5A4). Context thin, 0.5–1.2 mm thick in the center of the pileus, fibrous, pale yellow to pale orange (3A3–5A3), unchanging when bruised. Hymenophore decurrent, distant to subdistant, well developed, gill-liked ridges 1–1.5 mm high, mostly forked at margin, greyish yellow to greyish orange (3B5–5B5), unchanging when bruised. Stipe 30–50 mm long, 2–3 mm thick, subcylindrical, slightly tapering downward, smooth or with faintly scaly, hollow, concolourous to pileus. Odour none, Taste mild.

Basidiospores (100/4/4) (6–)6.5–8.5(–9) × (4.5–)5–6 μm, L_m_ × W_m_ = 7.55(±0.61) × 5.56(±0.34) μm, Q = (1.2)1.27–1.5(1.6), Q_m_ = 1.34 ± 0.09, elliptical to elongate elliptical. Basidia 37–74 × 7–9 μm, clavate, 5–6-spored, slender, narrowly clavate; sterigmata 5–7 μm long. Hymenophoral trama subparallel to regular, composed of colourless and branched hyphae, 4–10 μm wide, septate. Pileipellis a cutis, composed of ascending to erect and occasionally branched hyphae, 3–12 μm wide; terminal cell 38–120 × 7–10 μm, mostly cylindrical to subclavate, thin-walled. Stipitipellis a cutis of cylindrical, parallel hyphae, 3–8 μm wide, branched, septate, mostly encrusted with golden reflective substance. Stipe trama with hyphae 9–15 μm wide, clamped, septate. Clamp connections abundant in all tissues.

Habitat and distribution—Growing in groups or gregariously under mixed forests that are dominated by *Keteleeria* sp. and *Picea* sp. Currently known from southwest China.

Specimen examined—CHINA. Guizhou Province, Qiannan Buyi and Miao Autonomous Prefecture, Longli County, bought from Guanyin village mushroom market, 1 July 2020, alt. 1080 m, Ming Zhang (GDGM80788); Guiyang City, bought from a mushroom market, 1 July 2020, alt. 1080 m, Ming Zhang (GDGM80824); Longli County, Longjiashan National Forest Park, alt. 1000 m, 5 July 2020, Ming Zhang (GDGM80885).

Notes—*Cantharellus sinominor* is one of the most commonly documented *Cantharellus* species in subtropical coniferous forests in southwest China, and it can be found in local wild edible mushroom markets. In the field, *C. sinominor* is easily confused with *C. minor*, a species with small yellow basidiomata. The molecular phylogenetic analyses showed that they are closely related, but independent species. *Cantharellus minor* differs by the presence of its glabrous, bright yellow-orange to orange pileus, that is usually fading to pale orange-buff or pale orange, and its relatively large basidiospores (8–11 × 5–7 μm) [51,52,53]. Additionally, *C. minor* is reportedly associated with oaks and other hardwoods, whereas *C. sinominor* is associated with coniferous trees. 

*Cantharellus parvoflavus* M. Herrera, Bandala & Montoya, that is recently described from Mexico, is similar to *C. sinominor*. However, the former presents with a bright yellow-orange pileus with appressed fibrils at the centre, relatively narrow basidiospores (6–9 × 4.5–5 µm), and narrow hymenophoral trama hyphae (4–5 µm in diameter) [30]. 

*Cantharellus alboroseus* Heinem. and *C. tenuis* Heinem., two small species that were originally reported in the Congo, are morphologically similar to *C. sinominor*. However, *C. alboroseus* belongs to subg. *Rubrinus* and differs by the presence of its bright orange to pale pink pileus, small basidiospores [7.1–7.7(7.9) × (4–)4.1–4.7(–5) µm], and the absence of clamp connections [17]. *Cantharellus tenuis*, may belong to the subg. *Cinnabarinus* and differs by the presence of its tiny, bright orange basidiomata and small basidiospores (7–8 × 5–5.7 µm) [3,17].

**Figure 14:**
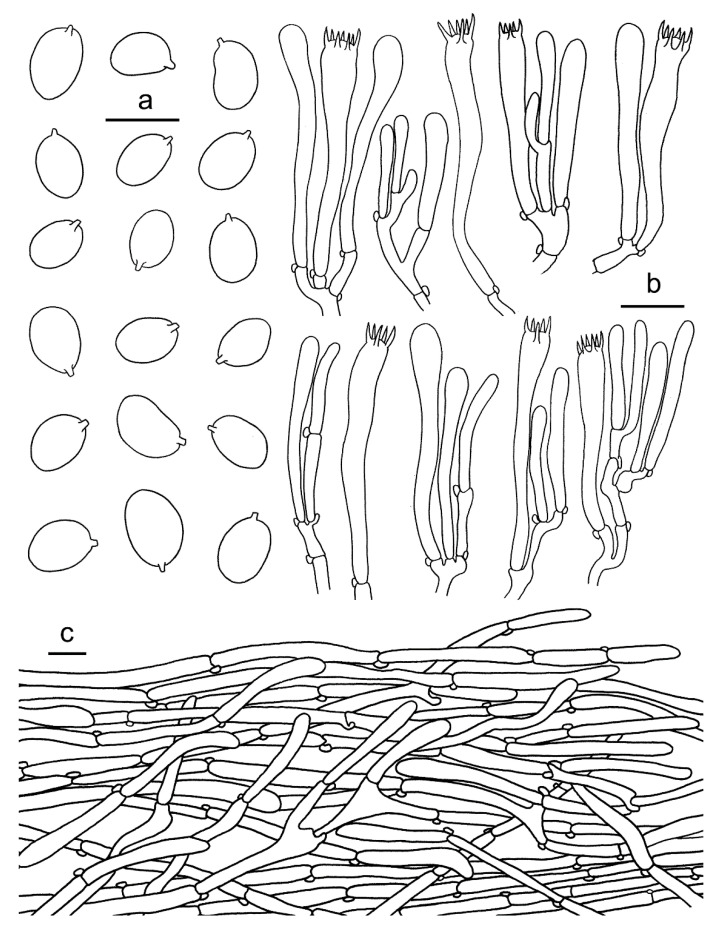
*Cantharellus zangii* (GDGM83193). (**a**) Basidiospores. (**b**) Basidia, basidiola and elements of the subhymenium. (**c**) Pileipellis. Bars: (**a**) = 10 μm; (**b**,**c**) = 20 μm.

***Cantharellus zangii*** X.F. Tian, P.G. Liu & Buyck, *Mycotaxon* 2012, *120*, 100; Figure 11h–i and Figure 14.

Synonym—*Cantharellus sikkimensis* K. Das, Buyck, D. Chakr., Baghela, S.K. Singh & V. Hofst., in Das, Hofstetter, Chakraborty, Baghela, Singh & Buyck, *Phytotaxa* 2015, *222(4)*, 273.

Basidiomata small-sized. Pileus 10–40 mm broad, convex when young, then applanate with center depressed at mature, surface dry, brown to dark brown (6E6–7E6, 6F6–7F6) at first, gradually fading to light brown to brownish orange (5C5–6C5, 5D5–6D5), glabrous, irregularly wrinkled, hygrophanous when wet, margin incurved and irregularly wavy. Hymenophore decurrent, subdistant, gill like, well developed, ridges 1.5–2 mm, composed of bifurcate and interconnected low veins, in particular toward pileus margin, veins usually less than 1 mm broad between ridges, light yellow (3A5), olive yellow (3C6), greyish yellow (4B6) to pale orange (5A4). Stipe 50–130 × 3–6 mm, subcylindrical, or gradually broader towards base, central, hollow, mostly twisted or longitudinally ridged or fluted, surface smooth, slightly waxy, brownish orange (5C5) to light orange (6A5) on upper half, greyish yellow (4B5) on lower half. Context 1–3 mm thick, greyish yellowish to olive brown (4B4–4D4), unchanging when bruised. Odour pleasant. Taste mild. 

Basidiospores (100/4/4) 8–11(–12) × 5.5–7(–8) μm, L_m_ × W_m_ = 9.25(±0.94) × 6.16(±0.45) μm, Q = (1.23)1.33–1.67(1.75), Q_m_ = 1.5±0.12, ellipsoid to sub-reniform, smooth, thin-walled, hyaline, sometimes with tiny oil drops. Basidia 70–90 × 8–11 μm, 5–6-spored, slender, narrowly clavate, with large number of vacuoles, sterigmata 5–8 μm long; basidiole 65–100 × 7–10 μm, numerous, clavate. Hymenial cystidia absent. Hymenophoral trama irregular to subparallel, composed of colourless and loose hyphae, 3–5 μm wide, branched, septate. Pileipellis a cutis, composed of repent to ascending and occasionally branched hyphae, 5–18 μm wide, subcylindrical; terminal cell 40–85 × 7–14 μm, mostly cylindrical to subclavate, thin-walled. Stipitipellis a cutis of cylindrical, parallel hyphae, 3–9 μm wide, branched, septate, mostly cylindric with clavate to subfusoid or rounded apices. Stipe trama with hyphae 9–15 μm wide, clamped, septate. Clamp connections abundant in all tissues.

Habitat and distribution—Growing in groups or gregariously under *Abies georgei* Orr and *A. densa* Griff. in subalpine coniferous forests or mixed forests. Known from southwest China and northern districts of India. 

Specimen examined—CHINA. Yunnan Province, Shangrila, Big Ravine, alt. 3030 m, 16 September 2007, Y.C. Li 537 (HKAS55743); Haba Snowy Mountains, alt. 3000 m, 30 September 2007, Feng Bang 182 (HKAS 55824); Bitahai National Natural Reserve, alt. 3850 m, 3 September 2020, Ming Zhang (GDGM82399, GDGM82389); Same location, 4 September 2020, Ming Zhang (GDGM83193, GDGM82374, GDGM83171, GDGM83173, GDGM83186).

Notes—As the type specimen of *C. zangii* was unavailable, two paratype specimens were carefully examined and compared with the newly collected specimens from the type locality in this study. The macro- and micro-morphological characteristics were observed to be well-matched. Therefore, data on new ITS, LSU, *tef*1, and *rpb*2 gene sequences of *C. zangii* that were derived from our newly collected specimens are provided in this study. 

The molecular phylogenetic analyses showed that *C. zangii* formed a distinct and well-supported lineage, and a sample named *C. sikkimensis* AB-2015 nested into the well-supported *C. zangii* lineage (100% BS and 1.00 BPP) in the multi-locus phylogenetic tree. *Cantharellus sikkimensis*, originally reported from India, is characterized by its dark brown pileus, light yellow hymenophore, long and hollow stipe, ellipsoid to sub-reniform basidiospores (8–11 × 5–7 μm) [39], which is highly consistent with *C. zangii* in morphology. Furthermore, a BLAST search that was based on the ITS sequence of *C. zangii* showed 99.14% identity percent to the sequence (accession no: KR001903) from the type specimen of *C. sikkimensis* AB-2015. Both of these species show distributions in subalpine coniferous forests and are associated with *Abies* plants. Thus, *C. sikkimensis* is a late synonym of *C. zangii*, and the distribution of *C. zangii* extends to the south of the Himalayas.

### 3.3. Key to Species of Subgenus Parvocantharellus in China


**1** Basidiomata white to yellowish-white............................................................................2**1’** Basidiomata not white, more obviously coloured with yellow to orange tinge......3**2** Pileus 20–50 mm wide, changing to yellowish when it is bruised; basidiospores 5.5–7.5 × (4) 4.5–6 μm.................................................................................................................
***C. albus***
**2’** Pileus 3–10 mm wide, unchanging in colour when it is bruised; basidiospores 4.5–7 × 4–5.5(–6.2) μm.....................................................................................................
***C. minioalbus***
**3** Pileus relatively larger, usually > 50 mm wide...................................
***C. appalachiensis***
**3’** Pileus smaller, usually < 50 mm wide.............................................................................4**4** Growing under coniferous trees.......................................................................................5**4’** Growing under broadleaf trees.......................................................................................8**5** Basidiospores 6–9 μm long, average length <8 μm......................................................6**5’** Basidiospores 8–12 μm long, average length >8 μm....................................................7**6** Pileus pastel yellow, light yellow to greyish-yellow, with a brownish-orange or reddish-brown center, glabrous or tomentosus at centre; basidiospores 6–8 × 4.8–6 μm; L_m_ × W_m_ = 7.05(±0.51) × 5.192(±0.34) μm...................................................................
***C. austrosinensis***
**6’** Pileus subtomentosus, greyish-yellow to greyish-orange, usually fading to light yellow to light orange toward the margin; basidiospores (6–)6.5–8.5(–9) × (4.5–)5–6 μm, L_m_ × W_m_ = 7.55(±0.61) × 5.56(±0.34) μm...................................................................
***C. sinominor***
**7** Pileus glabrous, bright yellow orange to orange, usually fading to pale orange-buff or pale orange; basidiospores 8–11 × 5–7 μm.................................................................
***C. minor***
**7’** Pileus light brown, brown to brownish-orange, glabrous; basidiospores 8–11(–12) × 5.5–7(–8) μm; distributed in subalpine region, associated with *Abies* sp..............
***C. zangii***
**8** Pileus very small, usually < 15 mm wide, greenish-yellow to yellow, well-developed gill-like ridges forked at the margin, basidiospores 6–7.5 × 4.8–5.5 μm..................................................................................................................................
***C***
**.**
***galbanus***
**8’** Pileus relatively large, usually > 15 mm wide...........................................................9**9** Basidiospores 6–7.5 μm long, average length < 7 μm, pileus yellow to yellowish-orange, hymenophore poorly-developed; basidiospores 6–7(–7.5) × (4.5)4.8–5.5(6) μm, average = 6.79× 5.07 μm...............................................................................................
***C. luteovirens***
**9’** Basidiospores 6–9 μm long, average length > 7 μm..................................................10**10** Pileus 20–32 mm wide, yellow to orange, hymenophore well-developed, basidiospores 7–8 × 5.2–6.5 μm, average = 7.44 × 6.04 μm......................................................
***C. luteolus***
**10’** Pileus 15–40 mm wide, light orange, hymenophore well-developed, basidiospores (6.5)7–9 × (4.5)5–6 μm, average = 7.95 × 5.51 μm.......................................
***C. aurantinus***



## 4. Discussion

In our multi-locus phylogenetic analyses, the ingroup sequences resulted in the formation of six main subgenera that is largely consistent with the most recent phylogenetic studies [1,2,8,9,26,34,46]. Thus, we adopted the treatment of Buyck et al. [1] and treated *C.* subg. *Parvocantharellus* as a monophyletic group, sister to the subg. *Cinnabarinus*. Apart from *C.* aff. *subcyanoxanthus*, the species of the subg. *Parvocantharellus* formed a well-supported (93% BS and 0.99 BPP) clade in the multi-locus phylogenetic tree. A total of nine species from China nested into this well-supported clade, including the seven new species that are described above: *C. aurantinus*, *C. austrosinensis*, *C. galbanus*, *C. luteolus*, *C. luteovirens*, *C. minioalbus*, and *C. sinominor*, and two previously reported species *C. albus* and *C. zangii*. In the phylogenetic analyses of the *tef*1 dataset, species in the subg. *Parvocantharellus* formed similar interspecific relationships in the multi-locus dataset, the seven new species were also well-supported, and further proved that the *tef*1 gene is suitable to determine the interspecific relationships for most species in *Cantharellus*. 

In this study, nine species were discovered from China, and they all belong to the *C.* section *Flavobrunnei*, which is characterized by the presence of medium-sized to extremely small basidiomata, a yellowish to brownish pileus, a long stipe, and abundant clamp connections. Phylogenetic analyses in our study all support the sect. *Flavobrunnei* as a monophyletic subclade in the subg. *Parvocantharellus*. In addition to the nine species that were introduced above, *C. appalachensis* and *C. minor* belonging to the subg. *Parvocantharellus* have also been reported in China [47,54,55,56]. *C. appalachensis* was proposed to demonstrate a geographically disjunct distribution from southeastern North America to eastern Asia [47]. However, only the LSU sequences of *C. appalachensis* that were derived from the Chinese samples were available, and these were not included in the phylogenetic analyses owing to the markedly low levels of genetic variation and the challenges that were encountered in distinguishing them among most of the species [57]. Thus, to determine the distribution of *C. appalachensis* in China, additional useful gene sequences from more samples are warranted. *C. minor* is considered a broad species and is widely distributed in most parts of China [54,55,56], including northeast, central, southern, and southwest China. Unfortunately, there were no specimen vouchers that were available for these records. The name “*Cantharellus minor*” is a collective name and has been misapplied to almost any small yellow *Cantharellus* species in China. The four new species *C. austrosinensis*, *C. galbanus*, *C. luteovirens*, and *C. sinominor* are easily misidentified as *C. minor* based on their morphological characteristics. However, phylogenetic analyses indicated that they represented five distinct species and *C. minor,* originally reported from North America under oaks and other hardwoods, can be distinguished by the presence of its large basidiospores (8–11 × 5–7 μm) [51,52,53]. In the present study, no samples of *C. minor* from China were detected in the phylogenetic analyses, and further studies with extensive sampling are warranted to determine the distribution of the species in China. The distribution of *C. minor* may be similar to that of *C. cibarius* in China. A recent study has shown that the distribution of *C. cibarius* is limited to northeast China, and the popular edible mushroom that is marketed in Yunnan, Guizhou, and Sichuan Provinces is, in fact, the native species *C. yunnanensis* W.F. Chiu [18].

Geographically, the species that are in the *C.* subg. *Parvocantharellus* are mainly distributed in the northern hemisphere and are especially diverse in Asia [16,35,38]. Except for *C. zangii* which has been reported in the subalpine regions of China and India, the remaining eight species were all reported in the subtropical and tropical regions of southern China, revealing an unexpectedly large number of new *Cantharellus* species in China, with a considerable number of species remaining to be discovered. Remarkably, in the phylogenetic trees, specimens that were from the northern hemisphere were clustered together in the well-supported sect. *Flavobrunnei* of subg. *Parvocantharellus*, representing a distinct northern hemisphere distribution clade. Meanwhile, three species, namely *C. avellaneus*, *C. congolensis*, and *C. subcyanoxanthus*, from the southern hemisphere formed the basal and sub-basal branches in the subgenus. *C. avellaneus* and *C. congolensis*, which belong to the sect. *Congolenses* Heinem., formed an isolated sub-basal clade in the subgenus and were characterized by their strongly blackening context and strong reaction with most macrochemical reagents. These distinct morphological characteristics make the species in this section easily distinguishable from *Flavobrunnei*, demonstrating a unique tropical African geographic distribution clade. *C. subcyanoxanthus*, which is characterized by its strong blue-violet-lilac to vinaceous basidiomata and yellow context, formed a monospecific sect. *Cyanomaculati* Buyck & V. Hofstetter, was located in the basal clade of subg. *Parvocantharellus*, but without significant support in the multi-locus phylogenetic tree. Based on molecular correlations, Buyck et al. [1] roughly divided the two sections in the subg. *Parvocantharellus*, but their morphological characteristics are not well-matched with the definition of the subg. *Parvocantharellus*. Thus, their distinct morphological characteristics and relatively independent phylogenetic positions may result in their assignment to a new subgenera level in the future as more related species continue to be discovered.

## Figures and Tables

**Table 1:** Information on the specimens that were used in the phylogenetic analyses. Sequences that were newly generated in this study are indicated in black bold.

Taxa	Voucher	Locality	GenBank Accession No.			Reference
			LSU	*tef*1	*rpb*2	
*Cantharellus afrocibarius*	BB 96.236	Zambia	KF294669	JX192994	KF294747	[5]
*C. afrocibarius*	BB 96.235	Zambia	KF294668	JX192993	KF294746	[5]
*C. albidolutescens*	BB 08.070	Madagascar	KF294646	JX192982	KF294723	[5]
*C. albidosquamosus*	PC0142511	Cameroon	MT002285	MT002270	MT004806	[33]
*C. alborufescens*	AH44223	Spain	KR677531	KX828816	KX828735	[34]
*C. albus*	HKAS107047	China	MT782542	MT776017	MT776014	[2]
*C. albus*	HKAS107048	China	MT782541	MT776016	MT776013	[2]
*C. albus*	HKAS107045	China	MT782540	MT776015	MT776012	[2]
***C. albus***	**GDGM56646**	**China**	**MZ605071**	**MZ613974**	**MZ614019**	**Present study**
***C. albus***	**GDGM81399**	**China**	**MZ605074**	**MZ613977**	**MZ614022**	**Present study**
***C. albus***	**GDGM81064**	**China**	**MZ605073**	**MZ613976**	**MZ614021**	**Present study**
***C. albus***	**GDGM77819**	**China**	**MZ605072**	**MZ613975**	**MZ614020**	**Present study**
*C. altipes*	BB 07.019	USA	KF294627	GQ914939	KF294702	[1]
*C. appalachiensis*	GRSM77088	USA	DQ898690	–	DQ898748	[35]
*C. appalachiensis*	BB 07.123	USA	KF294635	GQ914979	KF294711	[1]
***C. aurantinus***	**GDGM46278**	**China**	**MZ766517**	**MZ766560**		**Present study**
***C. aurantinus***	**GDGM46279**	**China**	**MZ766518**	**MZ766561**	**MZ766571**	**Present study**
***C. aurantinus***	**GDGM84974**	**China**	**MZ766521**	**MZ766564**	**MZ766572**	**Present study**
***C. aurantinus***	**GDGM84978**	**China**	**MZ766522**	**MZ766565**		**Present study**
***C. aurantinus***	**GDGM81889**	**China**	**MZ766519**	**MZ766562**	**MZ766574**	**Present study**
***C. aurantinus***	**GDGM81899**	**China**	**MZ766520**	**MZ766563**	**MZ766573**	**Present study**
***C. austrosinensis***	**GDGM60305**	**China**	**MZ605077**	**MZ613980**	**MZ614023**	**Present study**
***C. austrosinensis***	**GDGM79507**	**China**	**MZ605078**	**MZ613981**	**MZ614024**	**Present study**
***C. austrosinensis***	**GDGM81303**	**China**	**MZ605084**	**MZ613986**	**MZ614029**	**Present study**
***C. austrosinensis***	**GDGM81249**	**China**	**MZ605082**	**MZ613983**	**MZ614027**	**Present study**
***C. austrosinensis***	**GDGM80616**	**China**	**MZ605081**	**MZ613982**	**MZ614026**	**Present study**
***C. austrosinensis***	**GDGM80211**	**China**	**MZ605080**	**MZ613984**	**MZ614025**	**Present study**
***C. austrosinensis***	**GDGM81381**	**China**	**MZ605086**	**MZ613988**	**MZ614031**	**Present study**
***C. austrosinensis***	**GDGM81379**	**China**	**MZ605085**	**MZ613987**	**MZ614030**	**Present study**
***C. austrosinensis***	**GDGM81271**	**China**	**MZ605083**	**MZ613985**	**MZ614028**	**Present study**
***C. austrosinensis***	**GDGM82877**	**China**	**MZ605088**	**MZ613990**	**MZ614033**	**Present study**
***C. austrosinensis***	**GDGM80151**	**China**	**MZ605079**	**–**	**–**	**Present study**
***C. austrosinensis***	**GDGM81985**	**China**	**MZ605087**	**MZ613989**	**MZ614032**	**Present study**
*C. avellaneus*	1217/ER	Madagascar	KX857093	–	KX856997	[17]
*C. cerinoalbus*	AV 06.051	Malaysia	KF294663	–	KF294741	[1]
*C. cibarius*	GE 07.025	France	KF294658	GQ914949	KF294736	[1]
*C. cibarius*	BB 07.300	Slovakia	KF294641	GQ914950	KF294718	[1]
*C. cinnabarinus*	BB 07.053	USA	KF294630	GQ914984	KF294705	[1]
*C. cinnabarinus*	BB 07.001	USA	KF294624	GQ914985	KF294698	[1]
*C. congolensis*	1645/BB16.044	Saharan Africa	KX857102	KX857075	KX857006	[17]
*C. congolensis*	1676/BB16.123	Saharan Africa	KX857106	KX857078	KX857010	[17]
*C. aff. congolensis*	BB 06.176	Madagascar	KF294606	–	KF294680	[1]
*C. aff. congolensis*	BB 06.197	Madagascar	KF294608	–	KF294683	[1]
*C. curvatus*	BRNM:825749	South Korea		MW124390	–	[33]
*C. cyphelloides*	TNS F-61721	Japan	NG059027	–	–	[36]
*C. decolorans*	BB 08.278	Madagascar	KF294654	GQ914968	KF294731	[1]
***C. galbanus***	**GDGM86249**	**China**	**ZM766516**	**MZ766568**	**MZ766577**	**Present study**
*C. guyanensis*	1517/MR	Guyane	KX857095	KX857061	KX856999	[17]
*C. guyanensis*	1501/MRG07	Guyane	KX857094	KX857060	KX856998	[17]
*C. parvoflavus*	Montoya 5423	Mexico	MT371337	MT449706	–	[37]
*C. parvoflavus*	Herrera 204	Mexico	MT371338	MT449707	–	[37]
*C. parvoflavus*	Herrera 229	Mexico	MT371339	MT449708	–	[37]
*C. himalayensis*	DK-2010b	India	HM750917	–	–	[38]
*C. hygrophoroides*	HKAS80614	China	KJ004002	KJ004003	–	[23]
*C. ibityi*	BB 08.196	Madagascar	KF294650	GQ914980	KF294727	[1]
*C. koreanus*	1697/V. Antonin 14.115	Republic of Korea	–	KY271940	–	[17]
*C. koreanus*	1689/V. Antonin 13.136	Republic of Korea	–	KY271941	–	[26]
*C. lateritius*	BB 07.025	USA	KF294628	GQ914957	KF294703	[1]
***C. luteolus***	**GDGM44258**	**China**	**ZM766514**	**MZ766566**	**MZ766570**	**Present study**
***C. luteolus***	**GDGM60393**	**China**	**ZM766515**	**MZ766566**	**MZ766575**	**Present study**
***C. luteolus***	**GDGM86247**	**China**	**MZ766513**	**MZ766567**	**MZ766576**	**Present study**
***C. luteovirens***	**GDGM45899**	**China**	**MZ605095**	**–**	**–**	**Present study**
***C. luteovirens***	**GDGM80296**	**China**	**MZ605089**	**MZ613991**	**MZ614034**	**Present study**
***C. luteovirens***	**GDGM81395**	**China**	**MZ605093**	**MZ613995**	**MZ614037**	**Present study**
***C. luteovirens***	**GDGM81079**	**China**	**MZ605092**	**MZ613994**	**MZ614036**	**Present study**
***C. luteovirens***	**GDGM80672**	**China**	**MZ605090**	**MZ613992**	**MZ614035**	**Present study**
***C. luteovirens***	**GDGM80680**	**China**	**MZ605091**	**MZ613993**	**–**	**Present study**
***C. minioalbus***	**GDGM78910**	**China**	**MZ605098**	**MZ613999**	**MZ614043**	**Present study**
***C. minioalbus***	**GDGM78934**	**China**	**MZ605102**	**MZ614003**	**MZ614047**	**Present study**
***C. minioalbus***	**GDGM78883**	**China**	**MZ605096**	**MZ613997**	**MZ614041**	**Present study**
***C. minioalbus***	**GDGM78901**	**China**	**MZ605097**	**MZ613998**	**MZ614042**	**Present study**
***C. minioalbus***	**GDGM78916**	**China**	**MZ605100**	**MZ614001**	**MZ614045**	**Present study**
***C. minioalbus***	**GDGM78915**	**China**	**MZ605099**	**MZ614000**	**MZ614044**	**Present study**
***C. minioalbus***	**GDGM78926**	**China**	**MZ605101**	**MZ614002**	**MZ614046**	**Present study**
***C. minioalbus***	**GDGM78955**	**China**	**MZ605103**	**–**	**–**	**Present study**
***C. minioalbus***	**GDGM78997**	**China**	**MZ605104**	**–**	**–**	**Present study**
*C. minor*	BB 07.057	USA	KF294632	JX192979	KF294707	[1]
*C. minor*	BB 07.002	USA	KF294625	JX192978	KF294699	[1]
*C. pallens*	AH39124	Morocco	KX828804	KX828834	KX828755	[9]
*C. platyphyllus*	BB 98.126	Tanzania	KF294620	JX192975	KF294694	[1]
*C. pseudominimus*	JV 00.663	Portugal	KF294657	JX192991	KF294735	[1,5]
*C. rhodophyllus*	BB 16.126	Congo	MK422958	MG450695	–	[10]
*C. rhodophyllus*	BB 16.006	Congo	MK422957	MG450696	–	[10]
*C. romagnesianus*	AH44218	Spain	KX828807	KX828836	KX828757	[9]
*C. roseofagetorum*	AH44789	Georgia	KX828812	KX828839	KX828760	[9]
*C. sebosus*	BB 08.234	Madagascar	KF294652	JX192986	KF294729	[1]
*C. sikkimensis*	AB-2015	India	KP938966	–	–	[39]
***C. sinominor***	**GDGM80791**	**China**	**MZ605106**	**MZ614005**	**MZ614049**	**Present study**
***C. sinominor***	**GDGM80788**	**China**	**MZ605105**	**MZ614004**	**MZ614048**	**Present study**
***C. sinominor***	**GDGM80842**	**China**	**MZ605107**	**MZ614006**	**MZ614050**	**Present study**
***C. sinominor***	**GDGM80885**	**China**	**MZ605108**	**MZ614007**	**MZ614051**	**Present study**
*C. splendens*	BB 96.306	Zambia	KF294670	–	KF294748	[1]
*C. subalbidus*	OSC81782	USA	KX828814	KX828841	KX828762	[9]
*C. aff. subcyanoxanthus*	BB 98.014	Tanzania	KF294615	JX192973	KF294689	[1]
*C. subincarnatus subsp. rubrosalmoneus*	BB 06.080	Madagascar	KF294602	JX192963	KF294676	[1]
*C. symoensii*	BB 98.113	Tanzania	KF294619	JX192974	KF294693	[1]
*C. tabernensis*	BB 07.119	USA	KF294634	GQ914976	KF294709	[1]
*C. tabernensis*	BB 07.056	USA	KF294631	GQ914974	KF294706	[1]
*C. tenuithrix*	BB 07.125	USA	JN940600	GQ914947	KF294712	[1,40]
*C. texensis*	341/O7.120	USA	JN940601	GQ914987	KF294710	[1,40]
*C. texensis*	BB 07.018	USA	KF294626	GQ914988	KF294701	[1]
*C. tomentosus*	BB 98.060	Tanzania	KF294672	JX192995	KF294750	[1]
*C. tricolor*	BB 06.180	Madagascar	JN940604	JX192969	KF294682	[1,7]
***C. zangii***	**GDGM83171**	**China**	**MZ605113**	**MZ614012**	**MZ614056**	**Present study**
***C. zangii***	**GDGM83173**	**China**	**MZ605114**	**MZ614013**	**MZ614057**	**Present study**
***C. zangii***	**GDGM83186**	**China**	**MZ605117**	**MZ614016**	**MZ614060**	**Present study**
***C. zangii***	**GDGM82399**	**China**	**MZ605112**	**MZ614011**	**MZ614055**	**Present study**
***C. zangii***	**GDGM82389**	**China**	**MZ605110**	**MZ614009**	**MZ614053**	**Present study**
***C. zangii***	**GDGM83176**	**China**	**MZ605115**	**MZ614014**	**MZ614058**	**Present study**
***C. zangii***	**GDGM83181**	**China**	**MZ605116**	**MZ614015**	**MZ614059**	**Present study**
***C. zangii***	**GDGM82393**	**China**	**MZ605111**	**MZ614010**	**MZ614054**	**Present study**
***C. zangii***	**GDGM82374**	**China**	**MZ605109**	**MZ614008**	**MZ614052**	**Present study**
***C. zangii***	**GDGM83193**	**China**	**MZ605118**	**MZ614017**	**MZ614061**	**Present study**
***C. zangii***	**GDGM83228**	**China**	**MZ605119**	**MZ614018**	**MZ614062**	**Present study**
*Craterellus cornucopioides*	AFTOL-ID 286	USA	AY700188	–	DQ366287	[41]
*Cr. tubaeformis*	BB 07.293	Slovakia	KF294640	GQ914989	KF294717	[1,11]

## Data Availability

In Publicly available datasets were analyzed in this study. This data can be found here: [https://www.ncbi.nlm.nih.gov/; https://www.mycobank.org/; https://www.treebase.org/treebase-web/home.html, accessed on 28 July 2021].
